# Comprehensive characterization of extracellular vesicles produced by environmental (Neff) and clinical (T4) strains of *Acanthamoeba castellanii*

**DOI:** 10.1128/msystems.01226-23

**Published:** 2024-05-08

**Authors:** Elisa Gonçalves Medeiros, Michele Ramos Valente, Leandro Honorato, Marina da Silva Ferreira, Susana Ruiz Mendoza, Diego de Souza Gonçalves, Lucas Martins Alcântara, Kamilla Xavier Gomes, Marcia Ribeiro Pinto, Ernesto S. Nakayasu, Geremy Clair, Isadora Filipaki Munhoz da Rocha, Flavia C. G. dos Reis, Marcio L. Rodrigues, Lysangela R. Alves, Leonardo Nimrichter, Arturo Casadevall, Allan Jefferson Guimarães

**Affiliations:** 1 Departamento de Microbiologia e Parasitologia, Laboratório de Bioquímica e Imunologia das Micoses, Universidade Federal Fluminense, Niterói, Rio de Janeiro, Brazil; 2 Programa de Pós-Graduação em Microbiologia e Parasitologia Aplicadas, Instituto Biomédico, Universidade Federal Fluminense, Niterói, Rio de Janeiro, Brazil; 3 Departamento de Microbiologia Geral, Laboratório de Glicobiologia de Eucariotos, Universidade Federal do Rio de Janeiro (UFRJ), Rio de Janeiro, Brazil; 4 Programa de Pós-Graduação em Imunologia e Inflamação, Instituto de Microbiologia Paulo de Góes, Universidade Federal do Rio de Janeiro (UFRJ), Rio de Janeiro, Brazil; 5 Programa de Pós-Graduação em Doenças Infecciosas e Parasitárias, Faculdade de Medicina, Universidade Federal do Rio de Janeiro (UFRJ), Rio de Janeiro, Brazil; 6 Biological Science Division, Pacific Northwest National Laboratory, Richland, Washington, USA; 7 Instituto Carlos Chagas, Fundação Oswaldo Cruz, Fiocruz, Curitiba, Paraná, Brazil; 8 Centro de Desenvolvimento Tecnológico em Saúde (CDTS), Fiocruz, Rio de Janeiro, Brazil; 9 Instituto de Microbiologia Paulo de Góes, UFRJ, Rio de Janeiro, Brazil; 10 Rede Micologia RJ–Fundação de Amparo à Pesquisa do Estado do Rio de Janeiro (FAPERJ), Rio de Janeiro, Brazil; 11 Department of Molecular Microbiology and Immunology, Johns Hopkins Bloomberg School of Public Health, Baltimore, Maryland, USA; San Diego State University, San Diego, California, USA

**Keywords:** *Acanthamoeba castellanii*, extracelular vesicles, mPLEX, Neff, T4

## Abstract

We conducted a comprehensive comparative analysis of extracellular vesicles (EVs) from two *Acanthamoeba castellanii* strains, Neff (environmental) and T4 (clinical). Morphological analysis via transmission electron microscopy revealed slightly larger Neff EVs (average = 194.5 nm) compared to more polydisperse T4 EVs (average = 168.4 nm). Nanoparticle tracking analysis (NTA) and dynamic light scattering validated these differences. Proteomic analysis of the EVs identified 1,352 proteins, with 1,107 common, 161 exclusive in Neff, and 84 exclusively in T4 EVs. Gene ontology and Kyoto Encyclopedia of Genes and Genomes (KEGG) mapping revealed distinct molecular functions and biological processes and notably, the T4 EVs enrichment in serine proteases, aligned with its pathogenicity. Lipidomic analysis revealed a prevalence of unsaturated lipid species in Neff EVs, particularly triacylglycerols, phosphatidylethanolamines (PEs), and phosphatidylserine, while T4 EVs were enriched in diacylglycerols and diacylglyceryl trimethylhomoserine, phosphatidylcholine and less unsaturated PEs, suggesting differences in lipid metabolism and membrane permeability. Metabolomic analysis indicated Neff EVs enrichment in glycerolipid metabolism, glycolysis, and nucleotide synthesis, while T4 EVs, methionine metabolism. Furthermore, RNA-seq of EVs revealed differential transcript between the strains, with Neff EVs enriched in transcripts related to gluconeogenesis and translation, suggesting gene regulation and metabolic shift, while in the T4 EVs transcripts were associated with signal transduction and protein kinase activity, indicating rapid responses to environmental changes. In this novel study, data integration highlighted the differences in enzyme profiles, metabolic processes, and potential origins of EVs in the two strains shedding light on the diversity and complexity of *A. castellanii* EVs and having implications for understanding host-pathogen interactions and developing targeted interventions for *Acanthamoeba*-related diseases.

**IMPORTANCE:**

A comprehensive and fully comparative analysis of extracellular vesicles (EVs) from two *Acanthamoeba castellanii* strains of distinct virulence, a Neff (environmental) and T4 (clinical), revealed striking differences in their morphology and protein, lipid, metabolites, and transcripts levels. Data integration highlighted the differences in enzyme profiles, metabolic processes, and potential distinct origin of EVs from both strains, shedding light on the diversity and complexity of *A. castellanii* EVs, with direct implications for understanding host-pathogen interactions, disease mechanisms, and developing new therapies for the clinical intervention of *Acanthamoeba*-related diseases.

## INTRODUCTION

*Acanthamoeba castellanii* is a free-living amoeba (FLA) that is a vector and reservoir for various microorganisms and could have a role in the emergence or enhancement of the virulence of several pathogens through phagocytosis (1–8), which is similar to that of mammalian phagocytic cells (9–11). Recently, this FLA has gained recognition as a significant human pathogen, leading to challenging clinical cases ranging from keratitis to granulomatous amebic encephalitis (GAE), a rare but fatal disease (12–15). Despite this, difficulties in treatment, often requiring combinations of antibiotics, have been reported (16, 17).

Numerous studies have been conducted to elucidate the virulence characteristics of the *Acanthamoeba* genus, which currently encompasses 22 genotypes based on molecular analysis of the 18S rDNA gene (13, 18). Among these, the species *A. castellanii*, belonging to the genotype T4, is the most studied due to its wide geographical distribution (19–21), its high prevalence among clinical isolates from humans and animals opportunistic infections (12, 13), and its well-established intrinsic virulence properties (22, 23).

Given its pathogenic characteristics (24), studies with the genotype T4 often compare the virulence of clinical and environmental strains to understand the mechanisms involved during infection, invasion, and damage to the host tissues. The environmental strain known as Neff (ATCC 30010) is a widely used reference for characterizing virulence factors expressed by clinical isolates (25) and serves as a host model for interaction studies and the characterization of amoeba-resistant microorganisms, which encompass numerous important human pathogens, including the bacteria *Legionella pneumophila*, fungus from the genus *Fusarium* sp. and viruses such as adenoviruses and rotaviruses (3, 5, 26).

A variety of virulence factors have been identified in *Acanthamoeba*, including those involved in adhesion to host cells, such as the mannose-binding protein (27) and laminin-binding proteins (28); cytotoxic proteins like ecto-ATPases with molecular weights ranging from 62 to >300 kDa (29), and proteins aiding in host colonization, such as the neuraminidase (30). *Acanthamoeba* can secrete several factors independently of host cell contact to ensure survival and adaptation within the host, including superoxide dismutases (31), and hydrolases facilitating nutrient acquisition and host damage, including glycosidases (32), phospholipases (33, 34) and cysteine, metallo and serine proteases (35–38).

Regardless of the mechanism of damage, most of these virulence factors are transported to the extracellular milieu by classical and non-classical secretory pathways, with the latter also involving the production of extracellular vesicles (EVs) (36, 37). In T4 genotype strains of *A. castellanii*, these structures range from 50 to 700 nm and their contents vary drastically based on nutrient availability imposed on trophozoites (33, 39). EVs have also been reported to be produced by other genotypes (40). These EVs can damage distinct types of host cells and possess immunomodulatory properties during infection (33, 39, 41).

To determine the role of *A. castellanii* EVs in the metabolism, cell biology, and pathogenesis, we compared their morphology and composition in the environmental Neff strain and the clinical T4 (ATCC 50370) strain. Multi-omics techniques, such as the Metabolite, Protein, and Lipid Extraction (MPLEx) coupled to mass spectrometry (42) and RNA-sequencing (43) have been widely used in studies with biological samples to comprehensively understand how these structures might be involved in virulence, microbial adaptation, and gene expression regulation from a more dynamic and interactive perspective (44–46). While a significant number of studies in the literature have described the pathogenesis of *A. castellanii*, information comparing the metabolism, adaptation capacity and virulence of strains from distinct origins remains scarce. Therefore, characterizing metabolic pathways, molecules, and factors involved in their distinct pathogenic performances during the infectious process can provide valuable insights into the dynamics of metabolic pathways and molecules that characterize differences in pathogenic profiles between the environmental and clinical strains of this amoeba species. Thus, a detailed elucidation of the composition of *A. castellanii* EVs, critical virulence transmission factors, is potentially important for identifying new drug targets and development of novel therapies.

## MATERIALS AND METHODS

### Organisms and growth conditions

Two strains of *A. castellanii*, the environmental ATCC 30010 (Neff) strain and the clinical ATCC 50370 (T4) strain, both belonging to genotype T4 (Manassas, VA), were cultivated in peptone–yeast extract–glucose (PYG) medium containing 100 mM glucose, 0.4 mM CaCl_2_, 0.4 mM MgSO_4_, 2.5 mM Na_2_HPO_4_, 2.5 mM KH_2_PO_4_, 1 g/L sodium citrate, and 0.05 mM Fe(NH_4_)_2_(SO_4_)_2_, pH 6.5. *A. castellanii* was cultivated in culture flasks at 28°C until it reached confluence. Trophozoites were passaged every 48 h, resulting in a cellular viability of >99%.

### Isolation of EVs of *A. castellanii*

The EVs of *A. castellanii* from both Neff and T4 strains were obtained as previously described, following the MISEV2018 procedures (47, 48). Trophozoites were cultivated in 175 cm^2^ culture flasks containing 100 mL PYG, with a starting inoculum of 2.5 × 10^5^ amoebae/mL. After 48 h cultivation, about 1 L of cultures were centrifuged at 800 *g* for 10 min to pellet the trophozoites. The supernatants were additionally centrifuged at 15,000 *g* for 10 min. Sediments were discarded, and the new resulting supernatants were concentrated 20× by ultrafiltration in Amicon (cutoff = 10 kDa). The concentrated material was ultracentrifuged at 100,000 *g* for 1 h at 4°C (Beckman Optima LE-80K ultracentrifuge using the 70Ti Fixed-Angle titanium rotor) and washed with phosphate-buffered saline (PBS: 0.2 g/L KH_2_PO_4_. 0.2 g/L KCl. 2.4 g/L Na_2_HPO_4_ and 8 g/L NaCl; pH 7.2) by sequential centrifugations and resuspensions to isolate the EVs of *A. castellanii*. Protein concentrations were measured using the bicinchoninic acid (BCA) method with bovine serum albumin as standard, following the manufacturer’s instructions (ThermoFisher). Sterol concentrations were determined using the Amplex Red Cholesterol Assay Kit (ThermoFisher). Samples were frozen at −20°C until the moment of analysis.

### Morphological analysis of EVs from Neff and T4 strains of *A. castellanii*

A combination of methods was used to characterize the physical properties of EVs of *A. castellanii*. EVs from both strains underwent evaluations via transmission electron microscopy (TEM). For this, EV preparations were fixed for 2 h at RT with 2.5% glutaraldehyde, 4% paraformaldehyde, and 5 mM CaCl_2_ in 0.1 M sodium cacodylate buffer (pH 7.2). EVs were subsequently washed with PBS, followed by additional fixation for 1 h in 1% OsO_4_, 0.8% potassium ferrocyanide, and 5 mM CaCl_2_ in cacodylate buffer. EVs were ultracentrifuged at 100,000 × *g* for 1 h at 4°C. The resulting pellets were dehydrated with increasing concentrations of ethanol (ranging from 50% to 100%), clarified with acetonitrile, and infiltrated with araldite-Epon resin. Grids were visualized in a Jeol 100CX transmission electron microscope (JEOL, Japan) and the diameter of the EVs was calculated using ImageJ software (Bethesda, MD). Furthermore, the dimensions and concentrations of the EVs were determined using a NanoSight NS300 nanoparticle analyzer (NTA, Malvern Instruments). The instrument was equipped with a green 405 nm laser and an sCMOS camera at level 14, capturing 17.9 frames per second and a total of 1,071 frames. Viscosity was adjusted for 0.956–0.959 cP (water), and measurements were conducted at 21.8°C–22.1°C. Images and data were analyzed using the NTA 3.4 software (Build 3.4.4). The dimensions of the EVs were also assessed using dynamic light scattering (DLS), with a Quasi Elastic Light Scattering in a multi-angle analyzer particle size 90Plus/BI-MAS (Brookhaven Instruments Corp., Holtsville, NY) as previously described (49). Additionally, the values of EVs’ dimensions obtained by TEM and nanoparticle tracking analysis (NTA) techniques were compared and the Pearson correlation indexes were determined, assuming a significative correlation of *P* < 0.05.

### Metabolite, Protein, and Lipid Extraction of EVs

Three independent biological replicates of EV preparations of each *A. castellanii* strain (Neff and T4) were subjected to MPLEx following the protocol outlined previously (42). This consisted of adding water, chloroform, and methanol for chemical extraction and partitioning of the molecules. The EVs were then ultracentrifuged at 100,000 × *g* for 1 h at 4°C, followed by resuspension in the same volume of water and 5 volumes of cold chloroform-methanol solution (2:1 [vol/vol]) at −20°C. Subsequently, samples were incubated for 5 min on ice, vortexed for 1 min, and centrifuged at 12,000 rpm for 10 min at 4°C. The upper phase containing hydrophilic metabolites and the lower phase containing lipids were collected in glass autosampler vials, for metabolomic and lipidomic analyses, respectively. Proteins were recovered in the interphase and washed by the addition of 1 mL of cold methanol (−20°C), vortexed for 1 min, and centrifuged at 10,000 × *g* for 10 min. The supernatants were discarded, and the resulting pellets were dried in a Labconco Centrivap (Labconco, MO, USA) for 5 min.

### Proteomic analysis of EVs of *A. castellanii*

Proteins extracted from Neff and T4 EVs were dissolved in 50 mM NH_4_HCO_3_ containing 8 M urea. Protein concentration in triplicates was measured by the BCA assay (ThermoFisher). Dithiothreitol was added to a final concentration of 5 mM to reduce the disulfide bonds at 60°C for 30 min. Then, samples were alkylated by adding iodoacetamide to a final concentration of 40 mM for 1 h at 37°C. Samples were further diluted 10-fold with 100 mM NH_4_HCO_3_ previous to the addition of CaCl_2_ to 1 mM final concentration. Proteins were digested with trypsin (at a 1:50 wt:wt trypsin to protein ratio) for 3 h at 37°C, followed by salts and reagents extraction in SPE columns (1 mL Discovery C18, Supelco, Bellefonte, PA) as previously described (50). Five hundred nanograms of digested peptides, dissolved in water, were loaded into a column (4 cm × 100 µm ID) packaged in-house with 5 µm C18 (Jupiter) and separated into an analytical column (70 cm × 75 μm ID) packaged with C18 particles (3 µm), connected to a nanoUPLC system (Acquity, Waters). Elution was performed with a gradient of acetonitrile/0.1% formic acid (solvent B) and water/0.1% formic acid (solvent A) over 2 h at a flow rate of 300 nL/min (51). The gradient was maintained at 1% solvent B for 15 min followed by linear increments of solvent B as follows: 19 min, 8% B; 60 min, 12% B; 155 min, 35% B; 203 min, 60% B; 210 min, 75% B; 215 min, 95% B; 220 min, 95% B. The eluents were analyzed in tandem by nanospray in an orbitrap mass spectrometer (Q-Exactive Plus, Thermo Fisher Scientific) using a scanning window of 400–2,000 m/z with a 70,000 resolution at 400 *m*/*z*. Data-dependent tandem mass spectra were acquired by high-energy collision-induced dissociation (32% normalized collision energy) for the 12 higher-intensity precursor ions with multiple loads. The dynamic exclusion function was set to exclude fragmented precursor ions for 30 s. Data acquired from liquid chromatography coupled to tandem mass spectrometry (LC-MS/MS) were processed by the Maxquant software (v.1.6.17) (52). Peptides were identified by searching for spectra against the sequences of *A. castellanii* obtained from the Uniprot database (https://www.uniprot.org/, downloaded in July 2021). Methionine oxidation and N-terminal acetylation of the protein were considered as variable modifications, in addition to carbamidomethylation of cysteine residues as fixed modifications. The tryptic digestion of both terminal peptides was considered, but two sites of lost cleavages were allowed within each peptide. The remaining parameters have been set to software default. Label-free quantification was used to compare protein abundances between different samples.

### Lipidomic analysis of EVs from the Neff and T4 strains *of A. castellanii*

Lipids were dissolved in pure methanol and loaded in a C18 column (CHS 3.0 mm × 150 mm × particle size of 1.7 µm, Waters Corporation) connected to a NANOAcquity UPLC system (Waters Corporation) previously described (53). Lipids were separated into a gradient of ACN/H_2_O (40:60) (solvent A) and ACN/isopropanol (10:90) (solvent B) containing 10 mM ammonium acetate. The gradient program was as follows: 40%–50% B in 2 min, 50%–60% B in 1 min, 60%–70% B in 9 min, 70%–75% B in 3 min, 75%–78% B in 2 min, 78%–85% B in 2 min, 85%–92% B in 3 min, 92%–99% in 3 min, and kept for 9 min to 99% B. The flow rate was set at 250 µL/min and the eluted species were directly analyzed in an orbitrap mass spectrometer (Velos with ETD, Thermo Fisher). The six most abundant precursor ions were submitted to CID and HCD fragmentations. Lipid species were identified and cured manually with dedicated LIQUID software (53). For quantitative analysis, intensity peaks were extracted with MZmine v2.0 (54).

### Gas chromatography analysis coupled to mass spectrometry for metabolomic analysis in Neff and T4 EVs *of A. castellanii*

Hydrophilic metabolites were derivatized with methoxyamine and N-methyl-N-(trimethylsilyl)-trifluoroacetamide before being analyzed in an HP-5MS column (30 m × 0.25 mm × 0.25 μm; Agilent Technologies, Santa Clara, CA) coupled with gas chromatography–mass spectrometry (GC–MS; GC 7890A/MSD 5975C, Agilent Technologies) system. The injection was performed in splitless mode at 250°C, with the temperature set at 60°C. The temperature was maintained for 1 min and continuously increased at 10°C/min to 325°C, where it was held for 5 min. The fatty acid methyl ester (SIGMA Aldrich) pattern was used as a calibrator for retention time. Recalibration, deconvolution, and data extraction were performed with a metabolite detector (55). The molecules were identified by correspondence with FiehnLib (56) and nist14 GC–MS libraries with manual curation.

### Quantitative analysis of EV content from Neff and T4 strains and data integration

Comparison of the detected levels of protein orthologues, lipids, or metabolites was performed by Student’s *t*-test, considering equal variance and two-tailed distribution, with *P* ≤ 0.05 values considered statistically significant. For comparative analyses, missingness was evaluated for each channel/sample, and proteins to be conserved for quantification were selected to contain at least 67% of complete values (2/3 samples) for a given condition, the overall missingness was evaluated after filtering. A heatmap depicting the proteins passing the Student’s *t*-test (*P* ≤ 0.05) was plotted, and the two clusters obtained (enriched in Neff versus enriched in T4) were saved in the statistics. An enrichment analysis was performed for these two clusters by a modified Fisher’s exact test (EASE test) (57–59), using the homemade Protein-Mini-On Package (https://github.com/GeremyClair/Protein_MiniOn). First, the statistically modulated proteins (count query) were annotated into gene ontology (GO) categories according to their molecular function, cellular localization, and biological process and also Kyoto Encyclopedia of Genes and Genomes (KEGG) annotations and normalized to the total number of detected protein in the EVs as the universe (to calculate the % query). Second, the total number of proteins belonging to the same annotated categories in the whole genome was normalized to the universe of total genome proteins (% universe). Fold enrichment of GO or KEGG categories was calculated by dividing the (% query)/(% universe). Pathways analysis on distinct clusters was further evaluated with DAVID (60) and blast2go (https://www.blast2go.com/), and pathways were manually constructed with Vanted v2.8.3 (61). For the lipid ontology enrichment analysis, we have used the Rodin R package, available for non-R users as a web interface called Lipid Mini-On (https://omicstools.pnnl.gov/shiny/lipid-mini-on/) (62). Lipids enriched in each EV were used to perform an enrichment analysis against a universe of 185 lipid species using the Fisher exact test. For the metabolomics enrichment analysis, we used the MetaboAnalyst web interface (https://www.metaboanalyst.ca/) (63) against a universe of 86 metabolites and the Fisher exact test. In all enrichment analyses, only *P* ≤ 0.05 were considered statistically significant.

### Sequencing and comparison of small RNAs within EVs from Neff and T4 strains of *A. castellanii*

A total of 100 µg of EVs were used for the RNA isolation with the miRNeasy kit (Qiagen) according to the manufacturer’s instructions. The DNA cleanup step was performed in all samples using the DNase protocol (RNase-free, Qiagen). For the quantification and analysis of the integrity of RNA, we used the Qubit fluorimeter (ThermoFisher) and bioanalyzer Agilent 2100, RNA 6000 peak, and RNA small kits (Agilent Technologies). For the sRNAs and RNAs of the EVs, the libraries were built using the Kit TruSeq small RNA (Illumina) according to the manufacturer’s instructions. The samples were prepared in three independent biological replicates. The RNAseq was performed on a HiSeq 2500 (Illumina, single-end 50 bp SR mid-output run).

### Analysis of sequencing data

The sequences generated in fastaq format were analyzed by CLC Genomics Workbench v 20.0 (Qiagen), using the genome of *A. castellanii* Neff strain as a reference (NCBI RefSeq assembly GCF_000313135.1). The sequences generated were trimmed for the removal of the internal adapter (TGGAATTCTCGGGTGCCAAGG, 3' trim). The parameters for alignment were set as follows: mismatch cost (2), insertion cost (3), deletion cost (3), length fraction (0.8), and similarity fraction (0.8). The statistical analysis applied was the Differential Gene Expression method using the CLC Genomics Workbench v 20.0 (Qiagen). The RNA-seq analysis was conducted with specific parameters: the strand setting was configured for both strands, the library type set was designated as bulk, and expression was computed for genes lacking a transcript (Yes). The trimmed mean of M values was used for library size normalization and the mapping settings, the EM (expectation–maximization algorithm) estimation algorithm was employed. The differential expression analysis was based on a multi-factorial statistical approach using the negative binomial Generalized Linear Model. The false discovery rate (FDR) adjusted *P*-value was utilized as a multiple-testing correction. The transcripts expression was described as Transcripts per Million. The criteria for identifying differentially expressed transcripts were set at a minimum of twofold change and an FDR equal to or below 0.05.

### Statistical analyses

Statistical analyses were performed using GraphPad Prism 8. Comparisons between two groups were made by the Student’s *t*-test and *P* ≤ 0.05 values were considered statistically significant. Each experiment was repeated at least three times.

## RESULTS

### Comparison of growth kinetics between the Neff and T4 strains of *A. castellanii*

The growth kinetics of the environmental Neff and the clinical T4 strains of *A. castellanii* were compared up to 72 h culture. Despite the initially faster growth of the Neff strain, no statistical difference was observed for the cell density at the time of EVs harvesting (48 h), with 1.2 × 10^6^ trophozoites/mL for the Neff and 9.8 × 10^5^ trophozoites/mL for the T4 strain (*P* = 0.63, Fig. S1).

### Morphological comparison of EVs from Neff and T4 strains of *A. castellanii* reveals distinct dimensions

**Fig 1:**
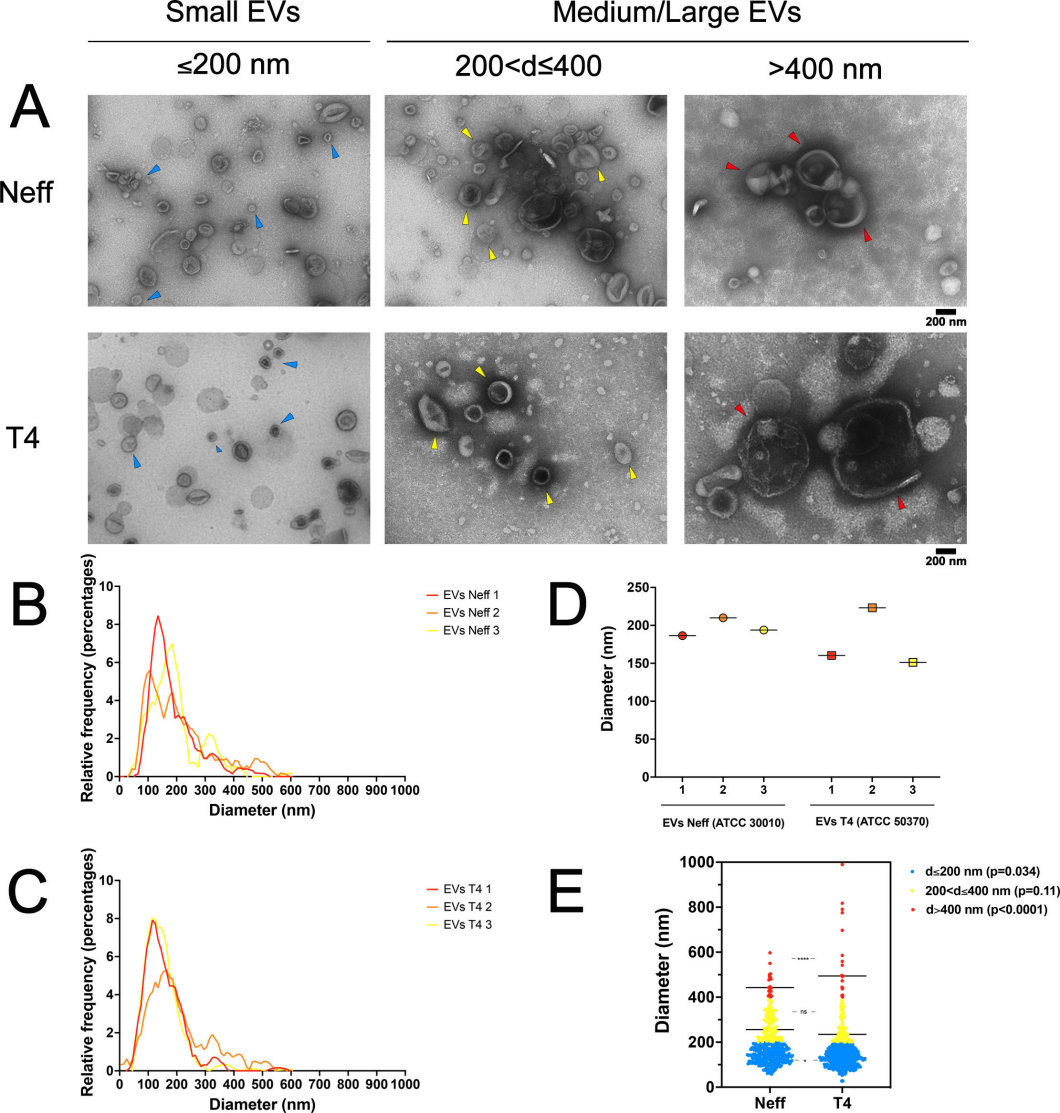
TEM images of EVs from the environmental Neff (ATCC30010) and clinical T4 (ATCC50370) strains of *A. castellanii* demonstrating variations in population sizes between strains. (**A**) EVs including exosomes and microvesicles from Neff and T4 strains. (**B and C**) Histograms depicting the frequency of EVs diameter by TEM (triplicates) for (**B**) Neff and (**C**) T4 strains, upon TEM and measurement in ImageJ. (**D**) Comparison of the mean EV diameter for Neff and T4 strains (each triplicate). (**E**) Scatter plot displaying EVs diameter of Neff and T4strains by TEM. Images captured at 46,000× magnification.

TEM was utilized for morphometric analysis assessing the diameter of EVs from environmental Neff and clinical T4 strains of *A. castellanii*. EVs were categorized based on the size as small (≤200 nm), medium (between 200 and 400 nm), and large EVs (>400 nm), as recommended by the MISEV 2018, its updated 2023 version, and other experts in the EV field (Fig. 1A) (47, 48, 64, 65). The histogram of size versus frequency for three independent EV preparations is shown in Fig. 1B and C for the Neff and T4 strains, respectively. A total of 410 Neff EVs were measured, with diameters ranging from 57.7 to 597 nm (mean diameter ± SD = 196.7 ± 12.0 nm). For the T4 clinical strain, a total of 594 EVs were evaluated, with diameters ranging from 27.4 to 990 nm (mean diameter ± SD = 178.2 ± 39.2 nm; Fig. 1C; *P* < 0.0001). Overall, a population of small EVs comprised 64.6% of Neff EVs compared to 77.3% of T4 EVs (139.3 ± 34.6 nm versus 130.3 ± 36.3 nm; *P* = 0.034), medium EVs constituted 30.2% and 19.5% (267.7 ± 51.6 nm versus 255.4 ± 52.4 nm; *P* = 0.11), and large EVs constituted 5.1% and 3.2% (458.1 ± 52.2 nm versus 560.9 ± 171.4 nm; *P* < 0.0001), respectively (Fig. 1D).

### Dimensions and size distribution of Neff and T4 EVs by NTA and DLS displayed similar values to TEM

**Fig 2:**
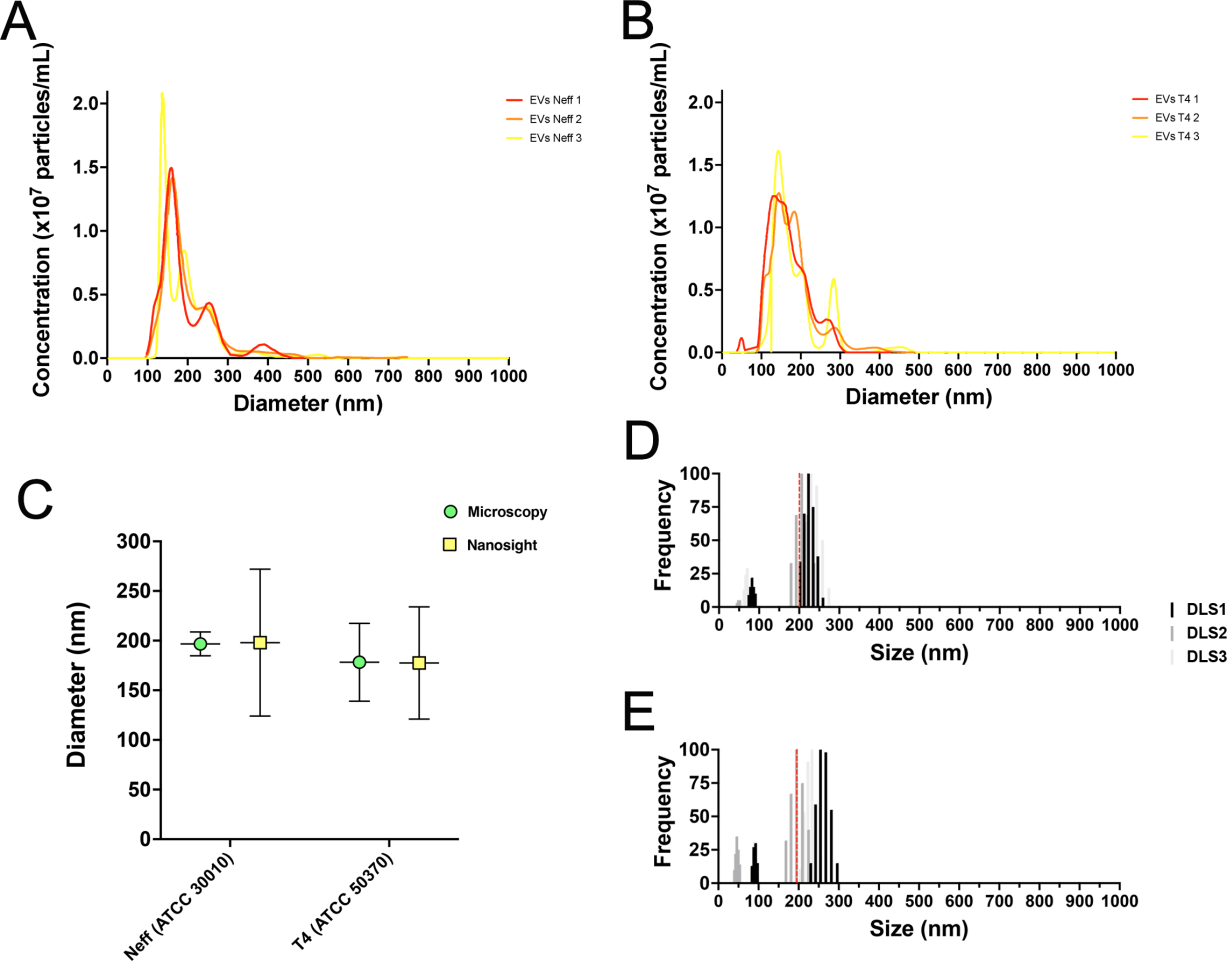
Frequency distributions of EVs diameters for the Neff and T4 strains of *A castellanii*, as determined by NTA and DLS. (**A and B**) Histograms presenting the frequency of EVs diameter using NTA for the (**A**) Neff and (**B**) T4 strains. (**C**) Mean diameter comparison of EVs from Neff and T4 strains obtained via TEM and NTA. (**D and E**) Frequency plots of EVs diameters of (**D**) Neff and (**E**) T4 strains using DLS. The red dashed lines indicate the mean diameter obtained for the EVs from each strain.

Assessment of the dimensions and concentrations of *A. castellanii* EVs from the Neff and T4 strains was performed using NTA. EVs isolation yielded similar concentrations as 1.1 × 10^9^ ± 2.1 × 10^7^ particles/mL and 1.3 × 10^9^ ± 1.0 × 10^7^ particles/mL, for the Neff and T4 strains, respectively. This resulted in approximately 4.5 × 10^8^ ± 8.2 × 10^6^ EVs/mL for the Neff and 5.1 × 10^8^ ± 4.1 × 10^7^ EVs/mL for the T4 strain at 48 h culture. Considering the cell concentrations measured by the growth kinetics, this renders about 388 ± 7 EVs/trophozoite for the Neff and 519 ± 41 EVs/trophozoite for the T4 strain. For both strains, EV populations corresponded to the size found in the literature representing small EVs (30–200 nm) and medium/large EVs (>200 nm), The Neff strain EVs had a size mean of 198.0 ± 74.0 nm considering the triplicate measurements (Fig. 2A), while the T4 strain EVs had a size mean of 177.5 ± 56.6 nm (Fig. 2B). When comparing both TEM and NTA techniques for measuring the EVs dimensions, we observed a significant correlation of EVs average (Fig. 2C, *P* = 0.98). Additional size evaluations were performed using the DLS technique. The Neff strain had a population of small EVs with a diameter of 45–90.8 nm and a population of small/medium EVs with diameter 179.6–273.9 nm, with an average diameter of 200.3 ± 56.6 nm (Fig. 2D). The T4 strain had a population of small EVs of 39.8–98.8 nm and small/medium EVs of 168.5–296.5 nm, with an average diameter of 195.6 + 70.9 nm (Fig. 2E).

### Assessment of protein and ergosterol levels of EVs from Neff and T4 strains of *A. castellanii*

**Fig 3:**
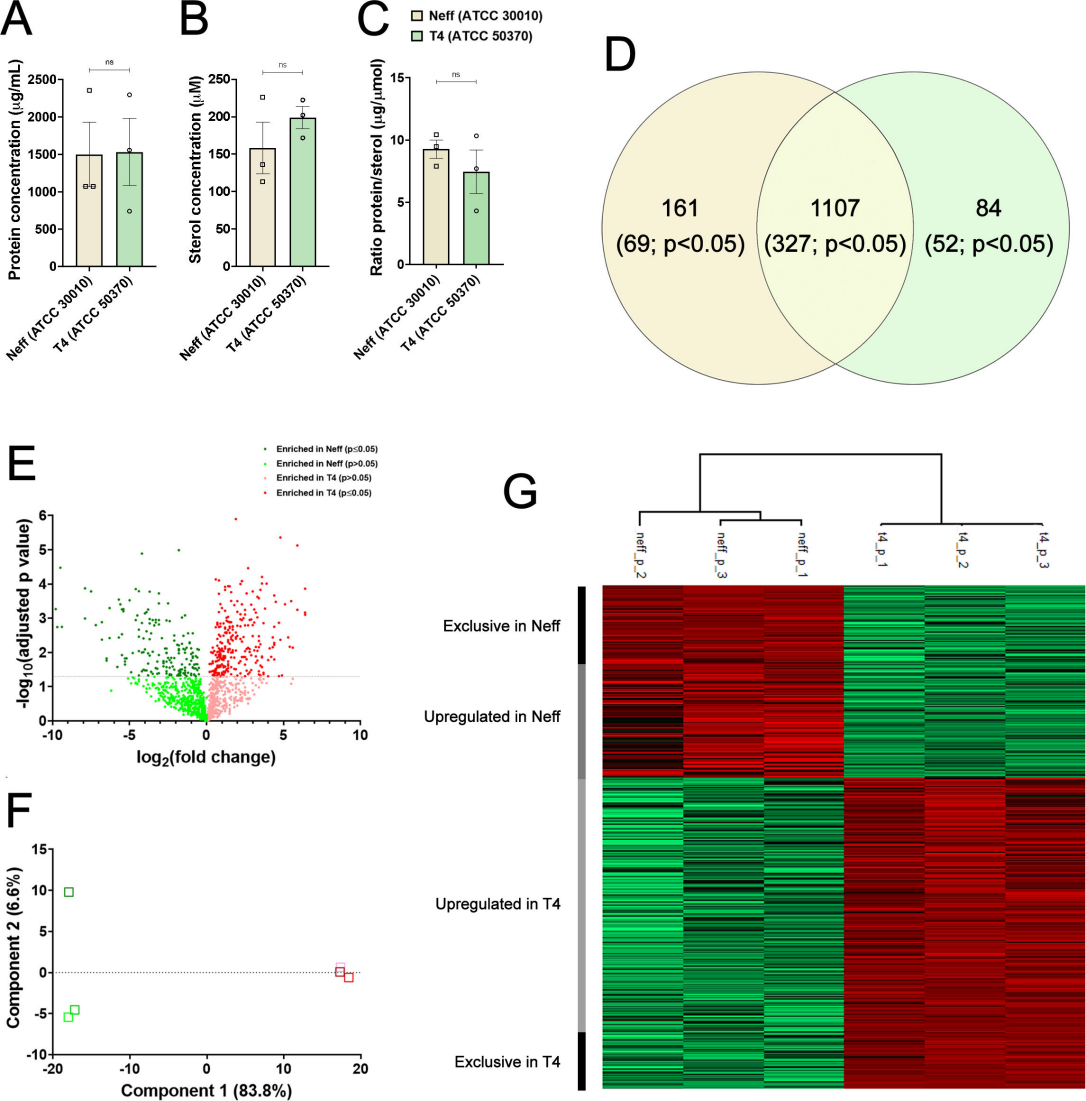
Biochemical and proteomic characterizations of EVs from Neff and T4 strains of *A. castellanii*. (**A**) The concentration of proteins found in the EVs of Neff and T4 strains. (**B**) Concentration of sterols found in the EVs of Neff and T4 strains. (**C**) Protein/sterol concentration ratio found in the EVs of the Neff and T4 strains. (**D**) Venn diagram illustrating the proteomic analysis of EVs of environmental (Neff) and clinical (T4) strains showing total, strain-specific, and shared proteins between the strains (and those with statistically significant differential expression, *P* < 0.05). (**E**) Volcano plot showing the magnitude of change (fold change T4/Neff) versus statistical significance (adjusted *P* value); dark green and red dots denote the differentially expressed proteins, more abundant in Neff and T4 EVs, respectively. (**F, G**) Conventional principal component analysis (PCA) demonstrating grouping similarity of proteomic data in EVs replicates from Neff (green squares) and T4 (red squares) strains and (**G**) a comprehensive heatmap illustrating proteins with significant differences in expression (*P* < 0.05, downregulated in green and upregulated in red) found in EVs from Neff and T4 strains. Analysis of EVs from each strain was performed in triplicate. The statistical analysis was performed using the *t*-test, with *P* < 0.05 considered statistically significant.

Protein and sterol levels were compared between Neff and T4 strains to initially discern differences in EV content (Fig. 3A through C). Although EVs from the clinical T4 strain showed slightly lower levels of protein and sterol (Fig. 3A and B, respectively), no differences were observed regarding the protein/sterol ratio compared to the Neff strain (Fig. 3C).

### Proteomic analysis reveals distinct protein cargos within EVs from Neff and T4 strains of *A. castellanii*

Proteomics analysis was used to compare the composition of EVs from Neff and T4 strains of *A. castellanii*. The results suggest potential shifts in global metabolic regulation and phenotypic behavior. A total of 1,352 proteins were identified in combining both strains, with 1,107 of these proteins commonly found in EVs of both strains, 161 exclusively found in Neff EVs, and 84 exclusively found in T4 EVs (Fig. 3D; Table S1). The volcano plot illustrates the significance levels and expression of each protein (Fig. 3E), while the principal component analysis (PCA) of the data set reveals distinct sample grouping based on EVs from different strains (Fig. 3F). Among the proteins identified, 448 exhibited statistically significant differential expression (*P* < 0.05), with 69 unique proteins found in Neff EVs, 52 in T4 EVs, and 327 proteins differentially expressed in both strains. Within the latter group, 102 proteins were upregulated in Neff EVs, and 225 were upregulated in T4 EVs (Fig. 3G).

**Fig 4:**
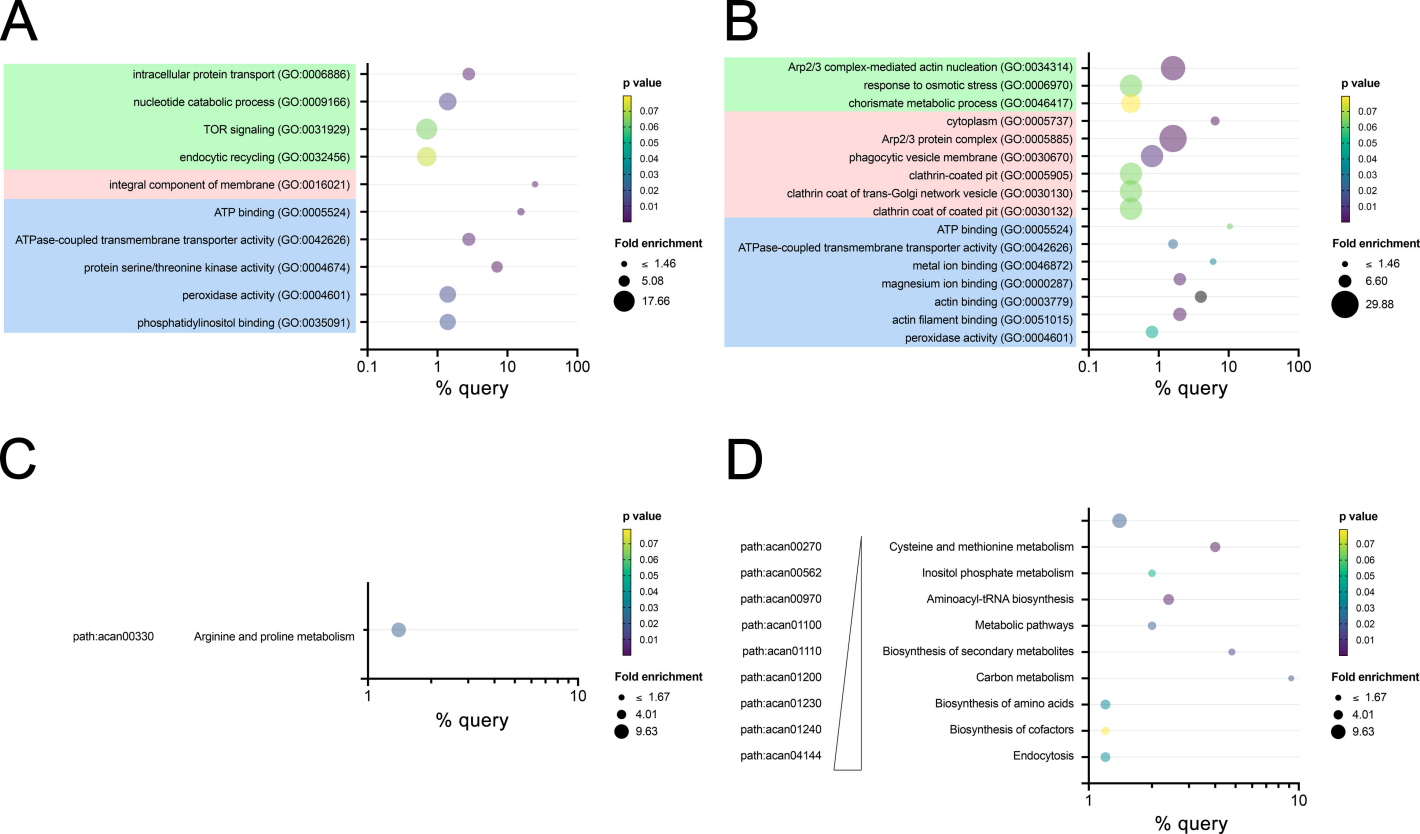
Enrichment analysis of proteins differentially regulated in EVs from Neff and T4 strains of *A. castellanii*. (**A and B**) Gene ontology enrichment analysis of the biological process (green), cellular component (pink), and molecular function (blue) for proteins more abundant (in EVs of the (**A**) Neff and (**B**) T4 strains of *A. castellanii*. Legends on the right panel (upper lane) for both (**A and B**) indicate the symbol size (fold change) (**C and D**) KEGG pathway enrichment analysis for proteins more abundant in EVs of the (**C**) Neff and (**D**) T4 strains of *A. castellanii*.

GO and KEGG mapping and enrichment analyses were conducted on the differentially expressed proteins in EVs from the Neff (171 protein) and T4 (277 proteins) strains of *A. castellanii* (Fig. S2), by comparing the percentage of detected proteins within each GO term to their frequency in the *A. castellanii* genome (Fig. 4). Both strains’ EVs showed enriched proteins associated with molecular functions like ATP binding (GO:0005524), ATPase-coupled transmembrane transport activity (GO:0042626), and peroxidase activity (GO:0004601). Additionally, EVs from the Neff strain displayed proteins specifically related to serine/threonine kinase activity (GO:0004674) and phosphatidylinositol binding (GO:0035091; Fig. 4A; Fig. S2A), whereas T4 EVs had proteins associated with metal ion (GO:0046872), magnesium ion binding (GO:0000287) and actin (GO:000379), and actin filament (GO:0051015) binding (Fig. 4B). In terms of biological process, Neff EVs showed enriched proteins involved in intracellular protein transport (GO:0006886), TOR signaling (GO:0031929), and endocytic recycling (GO:0062456). The majority of proteins belonged to the integral component of the membrane (GO:0016021) cellular component (Fig. 4A; Fig. S2A). Conversely, proteins in T4 EVs were associated with biological process such as Arp2/3 complex-mediated actin nucleation (GO:0034314), response to osmotic stress (GO:0006970) and chorismite metabolic process (GO:0046417), and cellular components such cytoplasmic (GO:0005737), phagocytic vesicle membrane (GO:0030670) and clathrin-coated groups (GO:0005905, GO:0030130, and GO:0030132) (Fig. 4B; Fig. S2B). While enrichment analysis was also performed for the KEGG function for both strains, insufficient data on enriched protein groups was found for Neff EVs, with a single KEGG term displaying enrichment (path: acan00330, arginine, and proline metabolism; Fig. 4C). In contrast, several KEGG terms exhibited enrichment for proteins in the clinical T4 EVs indicating distinct participation of proteins in various biosynthetic pathways and active endocytosis (path: acan04144; Fig. 4D).

### Distinct lipid profiles in Neff and T4 EVs

**Fig 5:**
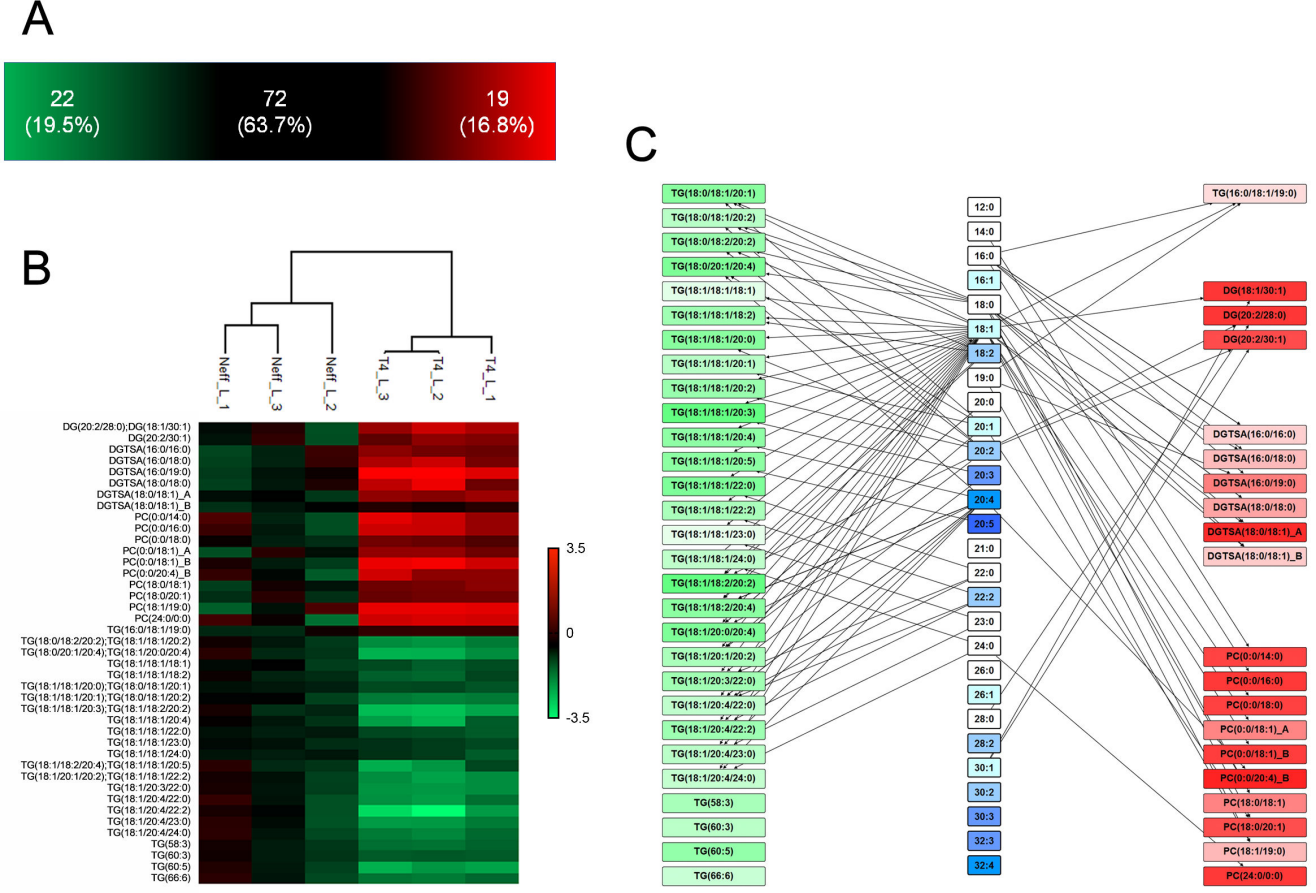
Analysis of total lipid composition obtained under the positive ionization mode of EVs from Neff and T4 strains *of A. castellanii*. (**A**) Number of detected lipids in positive ionization mode for EVs from Neff and T4 strains, with the statistically significant increased species (*P* < 0.05) number for each strain displayed in the green and red areas, respectively. (**B**) Heatmap of significant lipids (*P* < 0.05) grouped by lipid classes found in the positive mode analysis of EV from Neff and T4 strains of *A. castellanii*. Values were calculated after normalization of intensities by the mean values obtained for the environmental isolate Neff {log2[(sample)/(Neff average)]}. (**C**) Scheme denoting the fatty acid chains and unsaturation profiles found among the significant lipid classes obtained in the positive mode analysis of EVs from the Neff (green rectangles) and T4 strains (red rectangles). TG, triacylglycerol; DG, diacylglycerol; DGTSA, diacylglycerol-trimethylhomoserine; PC, phosphatidylcholine.

The lipidomic analysis encompassed a total of 185 species detected in the EVs of both strains. In the positive ion mode, 113 species were detected, with 22 species (19.5%, *P* < 0.05) enriched in Neff EVs, 72 species detected in both (63.7%), and 19 (16.8%) enriched in T4 EVs (Fig. 5A; Table S2; Fig. S3). The enriched species in Neff EVs were exclusively triacylglycerols (TGs; 22 species), whereas in T4 EVs the enriched species belonged to four distinguishing classes: phosphatidylcholines (PCs; 10), diacylglyceryl trimethylhomoserine/diacylglyceryl trimethyl-beta-alanine (DGTSA; 6), diacylglycerols (DG; 2), and TG (1) (Fig. 5B and C; Table 1; Table S2), showcasing a higher diversity in the latter group.

**TABLE 1:** Statistically significant **l**ipid species found for the positive and negative ionization mode of EVs from environmental (Neff) and clinical (T4) strains *of A. castellanii*^*a*^

Strain	Positive	Negative	Total
Neff	TG (22)	PE (19) PS (2)	43
T4	DG (2) DGTSA (6) PC (10) TG (1)	Cer (1) PA (2) PE (14)	36

^*a*^ TG, triacylglycerol; DG, diacylglycerol; DGTSA, diacylglycerol-trimethylhomoserine; PC, phosphatidylcholine; PS, phosphatidylserine; PE, phosphatidylethanolamine; Cer, ceramide; PA, phosphatidic acid.

**Fig 6:**
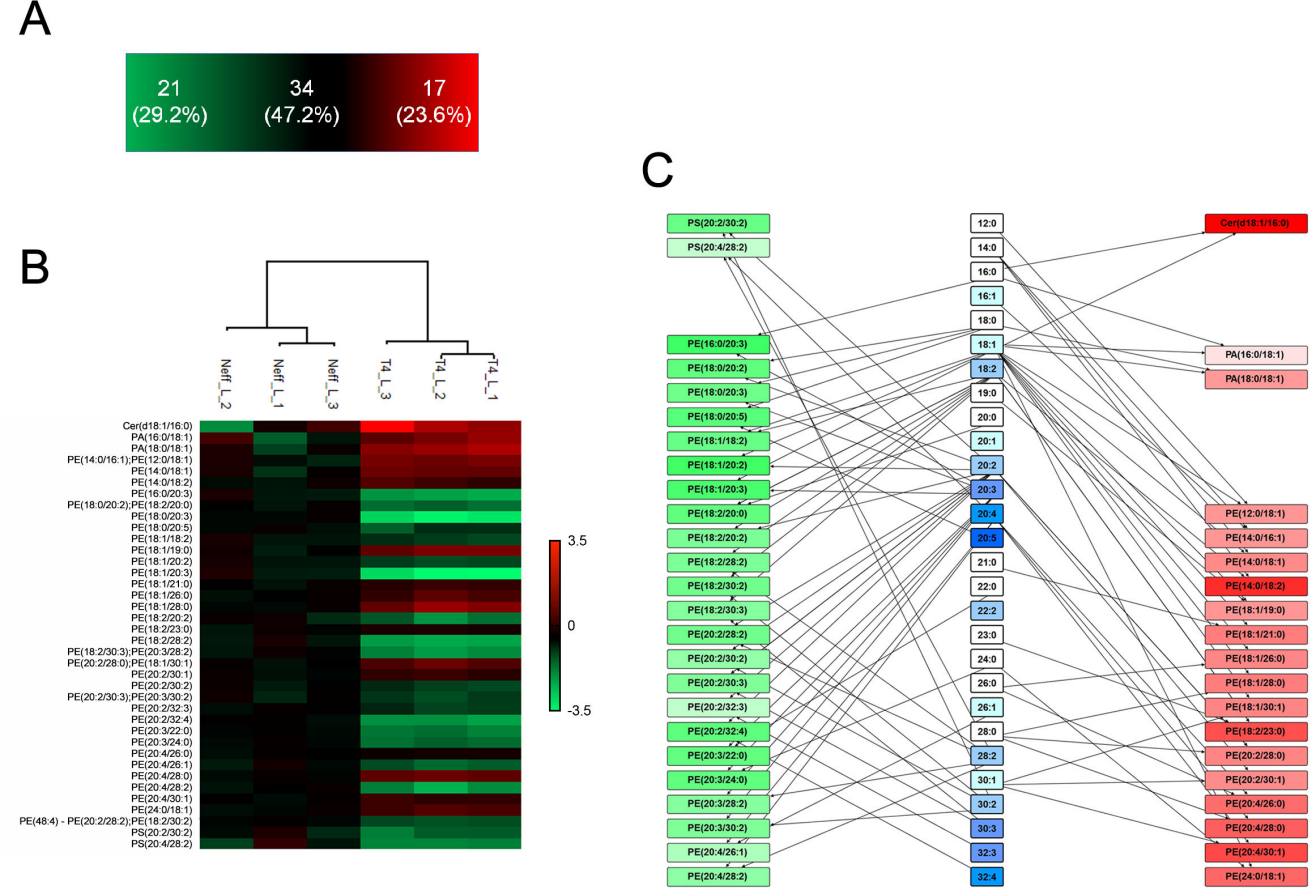
Analysis of total lipids composition obtained under the negative ionization mode of EVs from Neff and T4 strains *of A. castellanii*. (**A**) Number of detected lipids in negative ionization mode for EVs from Neff and T4 strains, with the statistically significant increased species (*P* < 0.05) number for each strain displayed in the green and red areas, respectively. (**B**) Heatmap of significant lipids (*P* < 0.05) grouped by lipid classes found in the negative mode analysis of EV from Neff and T4 strains of *A. castellanii*. Values were calculated after normalization of the intensities by the mean values obtained for the environmental isolate Neff {log2[(sample)/(Neff average)]}. (**C**) Scheme denoting the fatty acid chains and unsaturation profiles found among the significant lipid classes obtained in the negative mode analysis of EVs from the Neff (green rectangles) and T4 strains (red rectangles). PS, phosphatidylserine; PE, phosphatidylethanolamine; Cer, ceramide; PA, phosphatidic acid.

In the negative mode, a total of 72 lipids were identified in both strains’ EVs, with 21 species (29.2%, *P* < 0.05) enriched in the Neff EVs, 34 species detected in both strains (47.2%), and 17 species (23.6%) enriched in T4 EVs (Fig. 6A; Table 1; Table S3). Notably, despite the phosphatidylethanolamines (PEs) detection in both EVs, Neff EVs were enriched in PEs (19) displaying higher degrees of fatty acid unsaturation (Fig. 6B and C) and in phosphatidylserines (PSs; 2). Contrarily, T4 EVs exhibited enrichment in phosphatidylethanolamines (PEs; 14), phosphatidic acids (PAs) PA (16:0/18:1) and PA (18:0/18:1), and ceramide [Cer(d18:1/16:0)], indicating distinct regulation in lipid production between the two strains (Fig. 6C; Fig. S3B).

Overall, the enrichment analysis unveiled a more complex composition of T4 EVs (Table 2; Fig. S3A), with abundant glycerophospholipids such as PC, specific saturated fatty acids of 19:0 and 28:0 chains, and unsaturated 30:1 chain, PEs with a total number of chain unsaturation of 1 and TGs with a total chain carbon number of 53. On the other hand, Neff EVs were enriched with more unsaturated glycerolipids with a total number of chain carbon of 60, predominantly containing the 20:3 chain (Table 2; Fig. S3B).

**TABLE 2:** Lipid Ontology enrichment analysis of differentially regulated lipids in EVs from Neff and T4 strains of A. castellanii using the Lipid Mini-On.

	Lipid Mini-On enrichment	Classifier	Count query	Count universe	% query	% universe	*P*-value	FDR *q*-value	Fold change
Neff	Specific chain	20:3	7/96	7/377	7.29	1.86	0.0114	0.1196	3.93
	Specific chains by all	Glycerophospholipid with the chain 20:3	5/42	5/169	11.90	2.96	0.0287	0.4886	4.02
	Total chain carbon by all	Glycerolipid with a total number of chain carbon of 60	6/22	6/74	27.27	8.11	0.0272	0.2173	3.36
T4	Chain characteristics	Saturated	35/66	123/377	53.03	32.63	0.0020	0.0163	1.63
	Specific chain	30:1	3/66	3/377	4.55	0.80	0.0453	0.2569	5.71
	Specific chain	28:0	4/66	4/377	6.06	1.06	0.0198	0.2569	5.71
	Specific chain	19:0	4/66	5/377	6.06	1.33	0.0316	0.2569	4.57
	Category	Glycerophospholipid	26/36	92/185	72.22	49.73	0.0170	0.0341	1.45
	Main class	PC	10/36	21/185	27.78	11.35	0.0163	0.0570	2.45
	Subclass	LPC	7/36	10/185	19.44	5.41	0.0098	0.0392	3.60
	Total chain carbon by all	TG (with a total number of chain carbon of 53)	1/1	1/64	100.00	1.56	0.0308	10.000	64.00
	Total number of DB by all	Glycerophospholipid with a total number of unsaturation of 1	14/26	19/92	53.85	20.65	0.0023	0.0136	2.61
	Total number of DB by all	PE with a total number of chain unsaturation of 1	7/14	10/59	50.00	16.95	0.0146	0.0730	2.95
	Total number of DB by all	PE with a total number of chain unsaturation of 1	7/14	10/54	50.00	18.52	0.0328	0.1641	2.70

### Metabolite enrichment reveals distinct pathways involved in the EVs production in different *A. castellanii* strains

**Fig 7:**
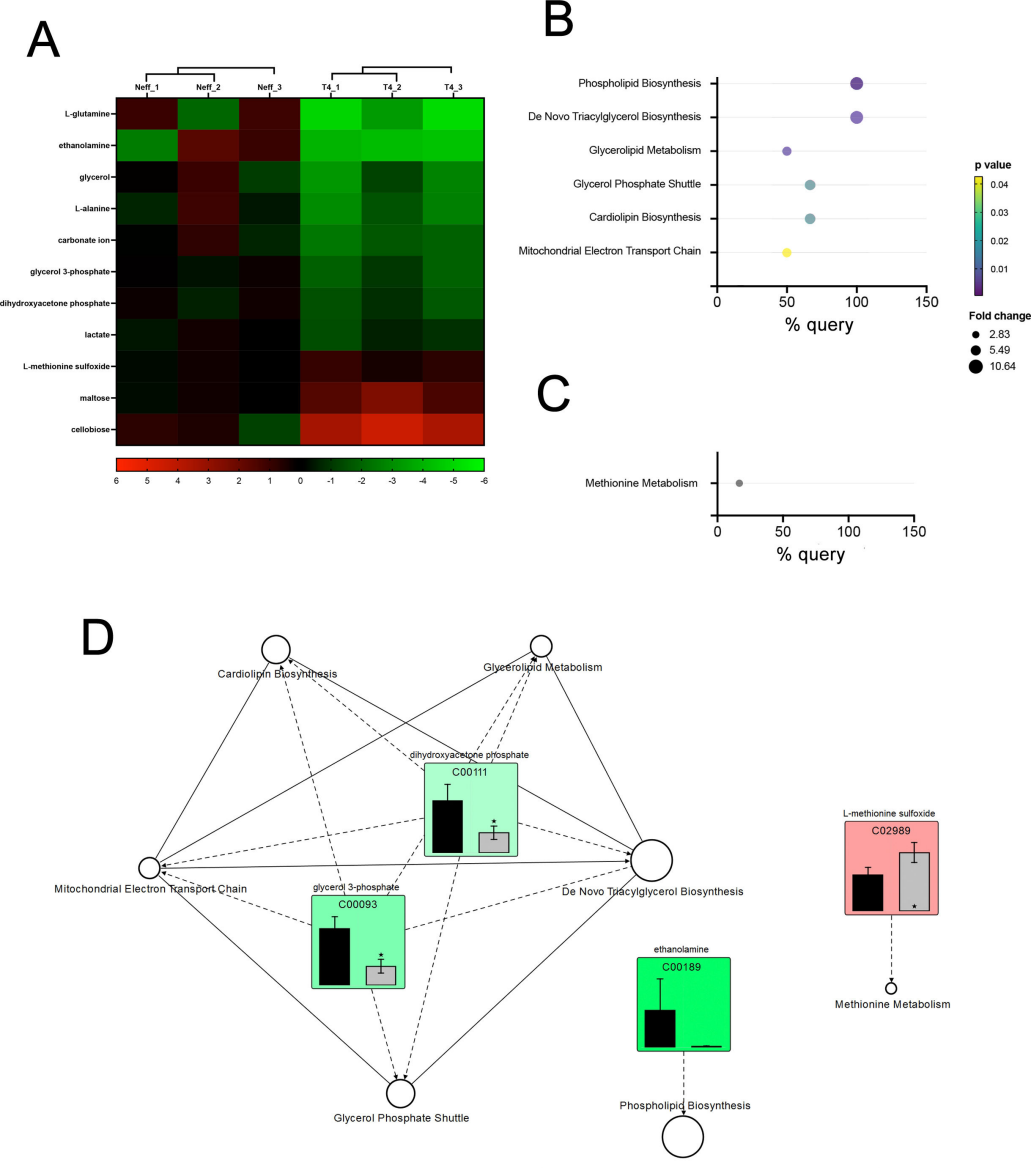
Analysis of metabolites for which significant statistical significances (*P* < 0.05) in EVs from the Neff and T4 strains *of A. castellanii*. (**A**) Heatmap of differentially abundant (*P* < 0.05) metabolites found in EVs from Neff and T4 strains. Values were calculated after normalization of the intensities by the mean values obtained for the environmental isolate Neff {log2[(sample)/(Neff average)]}. (**B and C**) KEGG pathway enrichment analysis for metabolites more abundant in EVs of the (**B**) Neff and (**C**) T4 strains of *A. castellanii*. (**D**) Diagram demonstrating the integration of lipid metabolic pathways of differentially abundant metabolites between the EVs from Neff (black bars) and T4 (gray bars) strains of *A. castellanii*. Green squares: metabolites more abundant in Neff EVs; red squares: metabolites more abundant in T4 EVs.

The metabolomic analysis of EVs from the Neff and T4 strains of *A. castellanii* identified a total of 86 metabolites (Table S4). Among these, 51 were more abundant in Neff EVs, while 35 were higher in T4 EVs. Considering the statistically significant differences in detection (*P* < 0.05), 11 metabolites were identified—eight predominant in Neff EVs and three in T4 EVs. Figure 7A displays a heat map illustrating the enriched metabolites. L-glutamine, ethanolamine, and glycerol were the three most prevalent species in Neff EVs, while cellobiose, maltose, and L-methionine sulfoxide were the most prevalent in T4 EVs. Enrichment analysis using the significant metabolites was performed based on the common metabolic pathway described in KEGG (https://www.genome.jp/kegg/pathway.html) (Fig. 7B and C). Phospholipid and triacylglycerols biosynthesis pathways were enriched in Neff EVs, along with the glycerolipid metabolism. In contrast, T4 EVs showed enrichment only in methionine metabolism. Fig. 7D presents a comprehensive diagram integrating detected species in EVs of both strains and statistically enriched pathways. Dihydroxyacetone phosphate and glycerol-3-phosphate, enriched in Neff EVs, are intermediates of the triacylglycerol and cardiolipin biosynthesis, glycerol phosphate shuttle and glycerolipid metabolism, along with the mitochondrial electron transport chain. Also, ethanolamine, more present in Neff EVs, is involved in the phospholipid biosynthesis, specifically of PE species. On the other hand, L-methionine sulfoxide, more abundant in T4 EVs, is an oxidation product of methionine.

### Detection of RNA molecules reveals distinct transcripts in EVs of different *A. castellanii* strains

**Fig 8:**
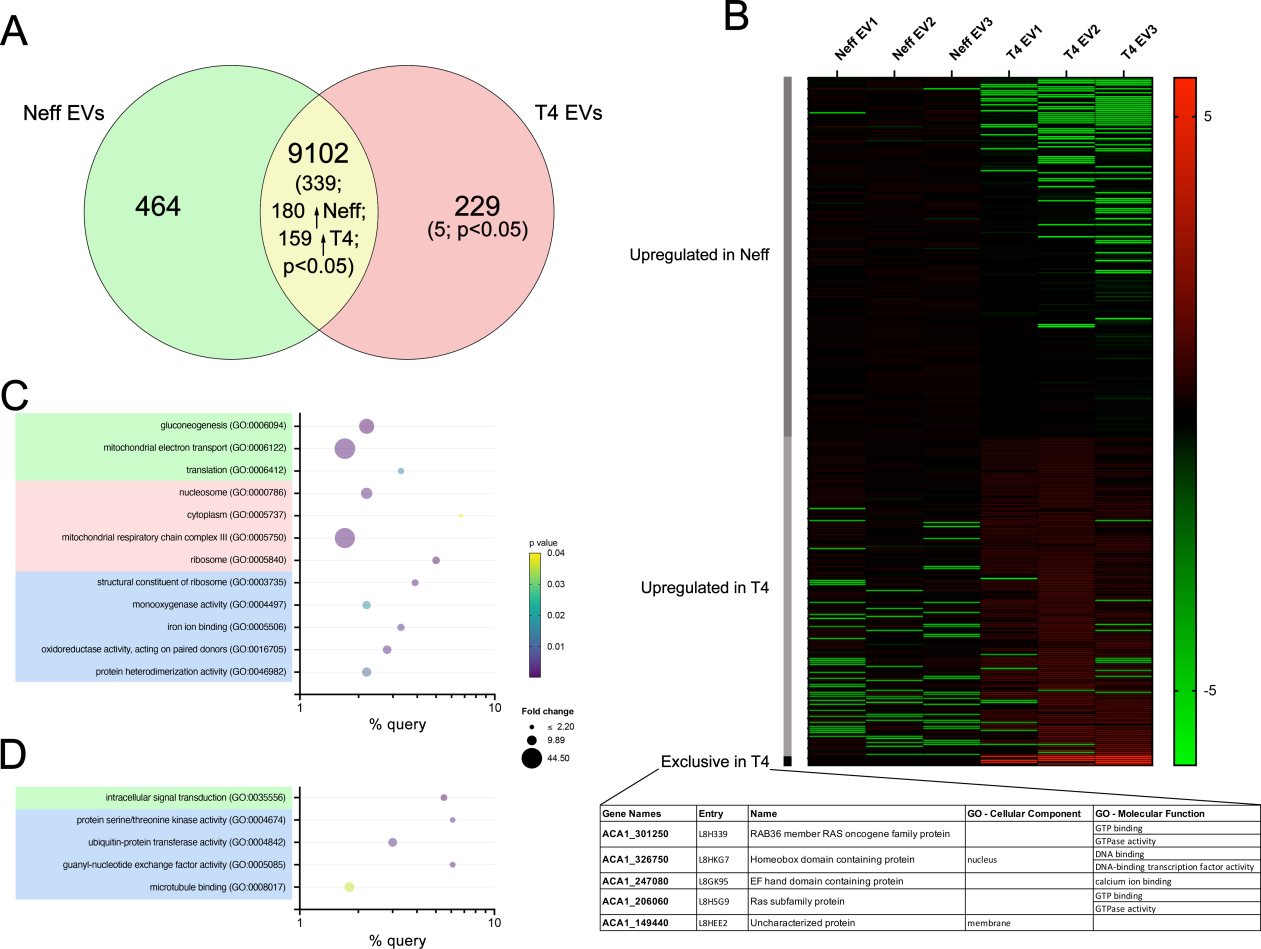
Analysis of the microRNA contents of EVs from Neff and T4 strains *of A. castellanii* using RNA-seq. (**A**) Venn diagram demonstrating the differential numbers of small and mRNAs transcripts detected in EVs from Neff and T4 strains *of A. castellanii*. (**B**) Heat map of the differentially regulated transcript (344), with 180 more abundant in Neff EVs, 159 more abundant in T4 EVs, and five exclusive transcripts found in T4 EVs. (**C and D**) Gene ontology enrichment analysis of the biological process (green), cellular component (pink), and molecular function (blue) for transcripts more abundant in EVs of the (**C**) Neff environmental and (**D**) T4 clinical strains of *A. castellanii*.

Most of the RNA molecules detected in the EVs ranged in size from 20 to 500 nt; however, we could also identify full-length mRNAs in the EVs. Analysis of the RNA contents of EVs via RNA-seq detected a total of 9,795 transcripts. Among these, 9,102 transcripts were present in the EVs of both strains of *A. castellanii*, with 339 transcripts showing differential abundance, as 180 were enriched in Neff EVs and 159 enriched in T4 EVs (Fig. 8A). Notably exclusive transcripts were detected in both EVs, with 464 found only detected in Neff EVs, and 229 in T4 EVs, and from this latter group, only five transcripts demonstrated statistical difference (Fig. 8A and B). Enrichment analysis of the pool of exclusive and differentially expressed transcripts in each EV indicates distinct transcript scenarios. In Neff EVs, transcripts involved in the biological processes of gluconeogenesis (GO:0006094) and translation (GO:0006412) were enriched, along with species typically found in the nucleosome (GO:0000786) and ribosome (GO:005840), contributing to various functions like structural constituent of ribosomes (GO:0003735), monooxygenase and oxidoreductase activity (GO:0004497 and GO:0016705, respectively) and protein heterodimerization activity (GO:0046982) (Fig. 8C). In the other hand, T4 EVs were enriched with transcripts involved in intracellular signal transduction (GO:0035556), along with molecular functions related to protein serine/threonine kinase activity (GO:0004674), ubiquitin-protein transferase activity (GO:0004842), guanyl-nucleotide exchange factor activity (GO:0005085) and microtubule binding (GO:0008017) (Fig. 8B and D).

### Data integration reveals distinct EV content from Neff and T4 strains of *A. castellanii* and different metabolic processes

**Fig 9:**
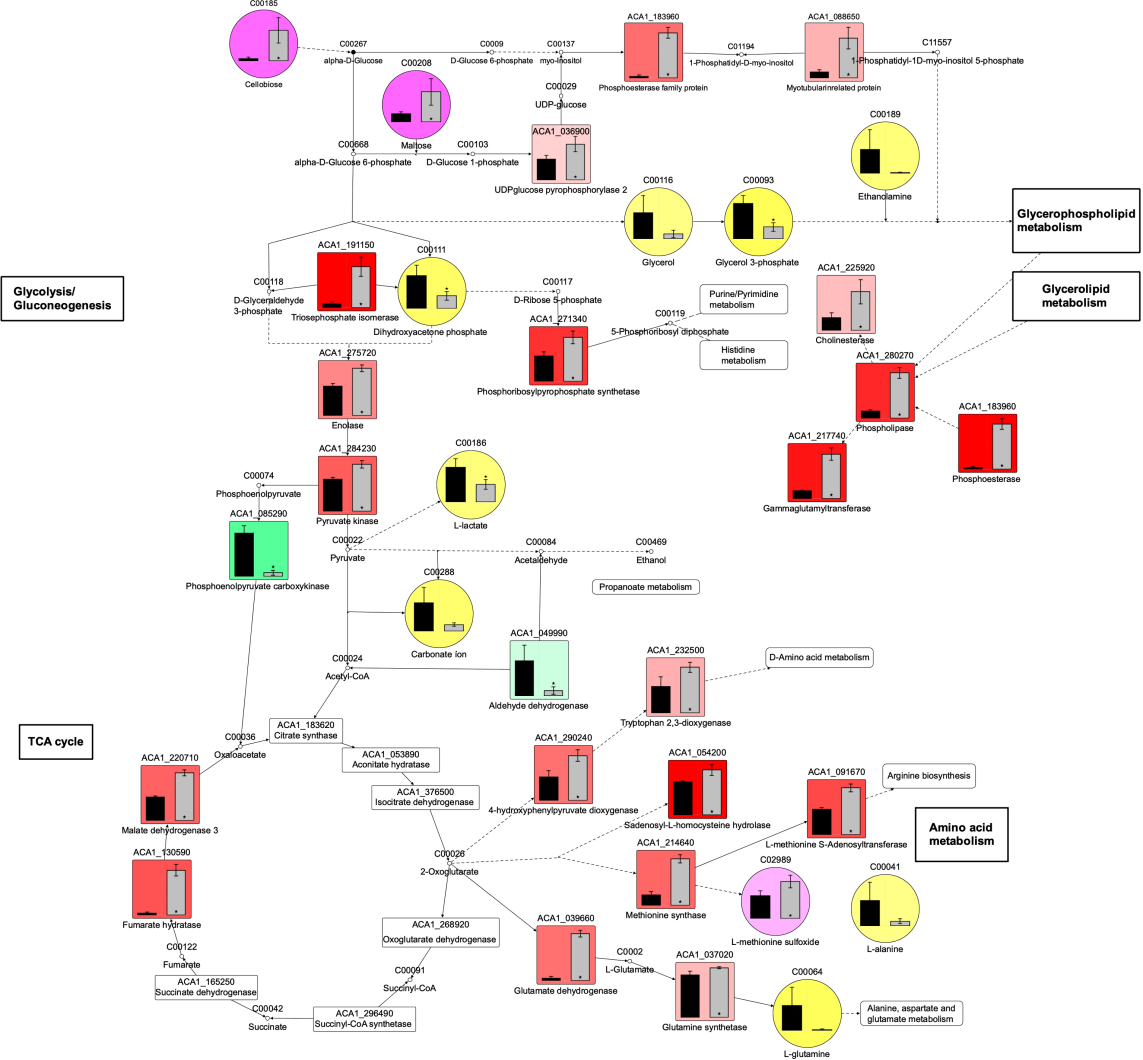
Diagram illustrating the levels of differentially regulated proteins (square graphs) and abundant metabolites (circles) participating in carbon metabolism and accessory metabolic pathways in the EVs of environmental Neff (black bars) and clinical T4 (gray bars) strains *of A. castellanii*. Green squares: proteins more abundant in Neff EVs; red squares: proteins more abundant in T4 EVs; yellow circles: metabolites more abundant in Neff EVs, and pink circles: metabolites more abundant in T4 EVs.

Enrichment analysis and data integration revealed significant differences in enzyme profiles related to central carbon metabolism in EVs from different *A. castellanii* strains (Fig. 9). This suggests variations in metabolic processes such as glycolysis, the TCA cycle, glycerophospholipid and glycerolipid metabolism and amino acid metabolism, which is more pronounced in the T4 strain, while the Neff strain may exhibit gluconeogenesis. Additionally, the higher levels of enzymes related to the synthesis of aminoacyl-t-RNA, nucleocytoplasmic transport, and mRNA surveillance pathway in T4 EVs, versus ribosomal biogenesis in Neff EVs indicate distinct mechanisms of transcription regulation (Fig. S4).

**Fig 10:**
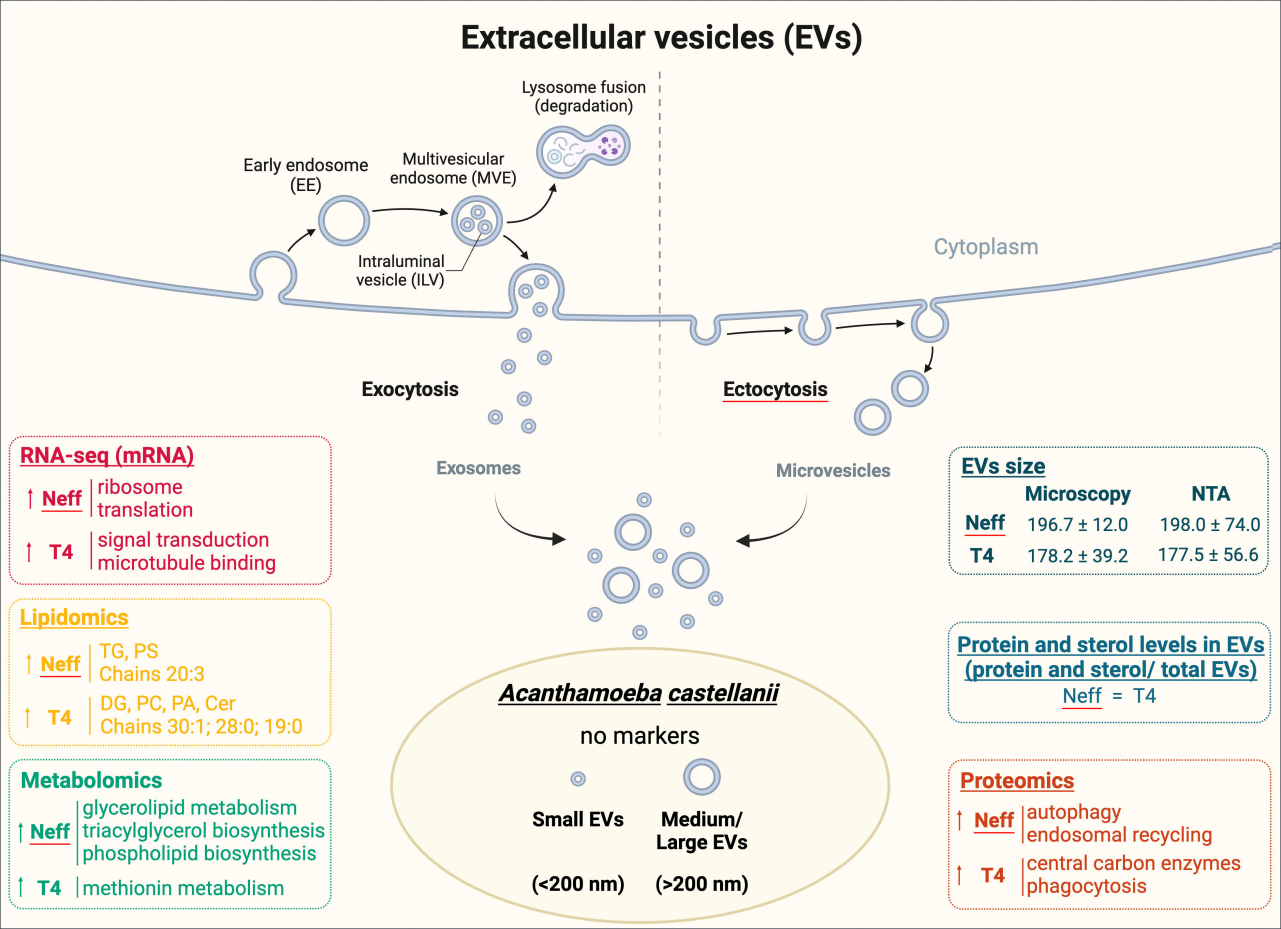
Diagram summarizing the results obtained throughout all techniques applied to compare the EVs composition of the environmental Neff and clinical T4 strains of *A castellanii*. As the literature lacks information regarding specific markers to define the origin and biogenesis of EVs from *A. castellanii*, we followed the guidelines recommended by the MISEV 2018 (48) and its updated 2023 version (65).

Moreover, the elevated levels of clathrin and Arp2/3 markers in T4 EVs, along with late endosomal markers (in contrast to early endosomal markers in Neff EVs), might suggest different origins of these EV pools. The increased mTor levels in EVs of the Neff strain imply a potential autophagy process and regulation in response to nutrient starvation, while T4 EVs show higher levels of phagocytic markers (Fig. S5). The overall observations of this study are summarized in Fig. 10.

## DISCUSSION

Advancements and application of new techniques have lately revealed the importance of EVs across various aspects of cell biology (66, 67). These nanostructures are now recognized not only for the simple cellular cargo transportation to the extracellular environment but also for cellular-level functions, including regulation of cellular metabolism, protein expression, and even more complex processes such as quorum sensing and populational density regulation (68, 69).

The vesicular secretion appears to be a common process in living organisms, representing a fundamental process for their viability, adaptation, and survival (36). In protozoa, non-conventional secretion via EVs has been described in trypanosomatids (70, 71), nematodes (72), and trematodes (73). In amoebas, opportunistic FLAs had already been characterized regarding the EV secretion: *Dictyostelium discoideum* (74, 75), *Acanthamoeba* sp. such as *A. castellanii* (37), the last pioneer study conducted by our group, and *A. culbertsoni* (76), and *Naegleria fowleri* (77–79). Additionally, EV secretion seems to be a conserved feature in human parasitic amoeba such as *Entamoeba histolytica* (80).

Closely analyzing published articles, the vast majority of EVs secreted by parasites are small EVs, typically ranging from 25 to 100 nm in diameter. These small EVs play roles in host-parasite interactions and have direct implications for the metabolic regulation of host cells due to the presence of microRNA components (72, 73, 81, 82). Moreover, they gain direct access to the host’s intracellular environment (83). For instance, *D. discoideum* has been reported to secrete nanovesicles characterized by TEM as three main populations: nanoEVs (<50 nm), directly involved in transit from the Golgi to the extracellular environment, and two small EVs populations (between 50 and 150 nm and >150 nm) (84).

TEM morphological analysis of the EVs from Neff (57.7–597 nm) and T4 (27.4–990 nm) strains of *A. castellanii* under axenic cultures overlapped previously reported values for the ATCC30234 in PYG (31.9 to 467 nm) and nutritional stress (33.7 to 303.2 nm) (37, 39). EVs’ dimensions were also confirmed by NTA and DLS. Both techniques rely on the characteristic Brownian movement of nanoparticles in suspension. However, DLS detection is based on the scattering of light depending on particle movement and size, which is sensed by detectors placed at different angles, while the NTA particle trajectory and the scattered light upon illumination are documented by a camera (85). Classically, the DLS drawback is the requirement of monodispersed suspensions; otherwise, data reliability is compromised as the measurement of particle dimensions is strongly influenced by larger particles that scatter more light (86). Then, DLS has been recommended for accurate determination of EV concentration, whereas NTA is for size determination with higher resolution (85, 87). Overall, all techniques show that the Neff strain had a less polydisperse EV population, as opposed to T4 EVs, which also had a population of larger EVs. Other studies also demonstrated the presence of EVs in other *A. castellanii* genotypes, such as the T5 genotype showing small EVs (50.29 ± 8.49 nm and 184.6 ± 50.80 nm) at 28°C, similar to both strains used in this study (40).

To date, reports on the compositional analysis of EVs from *A. castellanii* are scarce, primarily focusing on proteomics. Our group described the EV protein content, comparing their composition simulating different niches *A. castellanii* could be found *in vivo* (37). Under rich nutritional conditions, *A. castellanii* secreted EVs containing proteins involved in signaling pathways, suggesting their involvement in cell-to-cell communication as previously described for *D. discoideum* (88, 89). However, nutritional stress in the absence of protein sources caused a shift in EVs’ protein contents, with most proteins related to protein and amino acid and carbohydrate metabolism, proteases, cellular stress, and oxidative metabolism, with potential implications for nutrient acquisition, colonization of distinct host niches (37), and the amoebae differentiation between trophozoite and cyst (90).

The literature reports that pathogenic strains of the T4 genotype *of A. castellanii*, clinically associated with keratitis and GAE, express high levels of serine proteases, whereas other genotypes express metallo and cysteine proteases (37, 91). These are also secreted through EVs (36, 37, 41), and may pose direct implications in pathogenesis, promoting amoeba invasion digestion of extracellular matrix and mucosal protective barriers, evasion of the host’s immune response, host cell death and tissue damage, ultimately granting access of the parasite to the retina, where it can cause ocular keratitis or the central nervous system, causing encephalitis (92, 93).

Regarding the profile of other biomolecules and comparison among isolates within the T4 genotype, there is no report in the literature to date. This underscores the necessity for the application of multi-omic techniques such as the MPLEx and transcriptomics, coupled with integrational analysis, for the detection of EV components. We would like to reinforce that we sought to evaluate in this study the full composition at multiple molecular levels, including proteins, lipids, metabolites, and small RNAs, and comprehensive characterization of the *A. castellanii* EVs obtained from two distinct environmental and clinical strains upon standardized 48 h cultures and starting inoculum, to infer about distinct mechanisms involved in *A. castellanii* pathogenesis (94). We understand that variables such as inoculum, time of culture, and medium could impact the composition of EVs, but these should be evaluated and established singularly for each strain before a multifactorial comparison could be performed.

Proteomic analysis shed light on the distinct protein cargo within EVs from the Neff and T4 strains. For both strains, we identified a total of 1,352 proteins, with 1,107 proteins commonly found in both EVs, 161 proteins exclusively expressed in Neff EVs, and 84 proteins exclusively expressed in T4 EVs. These levels reflect a 10-fold higher number of proteins than previously reported by our group (37) and Liu et al. (39), due to the use of a significantly more sensitive technique/equipment. The proteomic findings were further substantiated by GO and KEGG mapping, with differences in the enriched proteins associated with specific biological and cellular processes, and molecular functions, suggesting potential variations in metabolic regulation and phenotypic behavior between the Neff and T4 strains. Overall, the proteins secreted and enriched in the EVs were primarily related to cellular trafficking and signaling pathways, but with distinct compositions depending on the strain. In accordance with our data, a prevalence of ATP-binding proteins was also found in the ameba *D. discoideum,* demonstrating their important role in amoebic survival (95). Additionally, a predominance of serine protease in the EVs of both but enriched in the clinical T4 strain was observed, in alignment with previous reports of their high expression in clinical strains of the T4 genotype (37, 41, 91).

EVs lipidomics has emerged as a novel tool to evaluate and compare alterations in the relative levels of lipid species in EVs which may vary by organism, cell type, growth conditions, and physiological state (96). Changes in the lipidome of EVs could provide information regarding EV origin and fate, trafficking, function, intracellular transport, and their stability in distinct environments, with direct implications for understanding differences in the cellular metabolism and their possible mechanisms of interaction with other cells, shedding light on their role in the pathogenesis of several diseases (97, 98). Recent studies have also demonstrated that some lipids are preferentially allocated or exclusive to specific populations of EVs, serving as potential biomarkers to understand the biogenesis and packing of these structures.

A comparison of the lipid composition of EVs from the Neff and T4 strains of *A. castellanii* demonstrated some striking differences, and overall environmental Neff EVs displayed a prevalence of unsaturated lipid species in comparison to mostly monounsaturated lipids for the clinical T4 strain. As these lipids have significant cell membrane structural functions, we can assume that this difference in arrangement between lipids modifies membrane permeability for each strain. Neff EVs had higher levels of TG glycerolipids, with enrichment of those with a total number of 60 carbons, whereas T4 EVs displayed more DGs and DGTSAs. These glycerolipids are commonly found in lipoproteins and lipid droplets, and the higher number of TGs in the environmental Neff EVs could indicate possible lipidic reserves, as opposed to its hydrolyzed DAGs in T4 EVs (99). Glycerophospholipids such as PC are usually more enriched in the cellular membranes than in EVs of eukaryotic cells. Their enrichment in the EVs of the T4 clinical strain as compared to the Neff EVs could indicate the distinct cellular origin of EEVs between strains. Consonantly, T4 EVs were enriched in Cer (d18:1/16:0), which could suggest the promotion of microEVs secretion by this strain, as also observed in other models (100, 101). Instead, Neff EVs were enriched in PS and polyunsaturated PEs, classical features of MVB-derived EVs, and exosomes that may also be involved in cell-to-cell communication and internalization of EVs by the recipient cells (102–104). However, the lack of specific markers to define the origin and biogenesis of the distinct populations of EVs, as exosomes or microEVs, for each strain used in this study, does not allow us to move forward at this stage in such characterization.

The metabolomic analysis revealed significant metabolite differences between the strains. Neff EVs displayed a similar profile to that of *E. histolytica*, which in a stress situation had a significant expression of glycerol and glycerol-3-phosphate, redirection of the glycolytic pathway, characteristics not observed for the clinical isolate T4. This indicates a difference in the regulation of metabolic pathways between strains (105). Elevated levels of ethanolamine in Neff EVs might also indicate a regulatory mechanism toward the synthesis of TG and PE, as part of the enriched pathways of glycerolipid metabolism, *de novo* triacylglycerol, and phospholipid biosynthesis.

The presence of RNAs in EVs might indicate their involvement in various genetic processes and regulation of cell physiology. Non-coding RNAs in EVs might display important regulatory functions upon delivery to the recipient cell, just as mRNA might also be translated into functional peptides once delivered, both ultimately shaping the transcriptome of the recipient cell (106, 107). In Neff EVs, transcripts related to gluconeogenesis (GO:0006094) might reinforce a metabolic shift, along with transcripts related to translation (GO:0006412), nucleosome (GO:0000786), and ribosome (GO:0005840), which might explain a populational gene regulation strategy. In contrast, in T4 EVs, proteins related to signal transduction (GO:0035556) might indicate a prompt response for environmentally-induced metabolic adaptation.

Upon analyzing the outcomes of multi-omic and morphology of EVs from the two *A. castellanii* strains, Neff and T4, distinct regulatory mechanisms governing metabolic pathways and EV genesis become apparent (Fig. 10). This distinction was notably evident in the differential expression observed between the strains concerning the diversity of lipid classes, with the EVs of the clinical strain T4 manifesting six distinct lipid classes compared to the three in the environmental Neff strain. Moreover, variations in unsaturation levels, notably observed in phosphatidylethanolamines, further delineated the unique lipid profiles characterizing each strain. This lipidomic variance is corroborated by the functional differences of proteins identified in the strains, aligning with specific metabolic processes. The Neff strain exhibits a higher concentration of proteins associated with autophagy and the regulation of nutrient deficiencies, highlighting a robust internal regulatory machinery. Conversely, the T4 strain shows enrichment of proteins involved in phagocytic processes, reflecting a pronounced focus on interactions with and adaptation to the external environment. This contrast is similarly observed in RNA analysis, demonstrating a heightened internal regulatory activity in Neff and greater adaptability to diverse metabolic pathways in T4.

In this broader context, we observed in general that the EVs of the Neff and T4 strains of *A. castellanii* have different compositions due to the distinct behavior of their generating cells in response to their respective environment. The environmental strain displayed a rigorous regulation of mechanisms involved in the internal machinery of the cell, while for the clinical strain T4 there was a greater presence of markers aimed at interaction with the external environment, as evidenced by phagocytosis markers. This divergence in behavior offers valuable insights into the different damage profiles the two strains cause during infection in a host.

Thus, our results reinforce the need for further multi-omic studies targeting EVs as critical pathogenic mechanisms thanks to their ability to carry molecules with high virulent potential to the external milieu. In addition, comparative analysis of EV composition across various strains enables the exploration of secretion pathways as potential pharmacological targets for the treatment *of A. castellanii* infections. With the application of increasingly integrative techniques, such as MPLEx used in the current work, we have been able to further advance the knowledge of the plasticity of metabolic pathways linked to the virulence of diverse pathogens, as is the case of *A. castellanii*.

## Data Availability

The metabolomic, proteomic, and lipidomic data are available at the MassIVE repository under the accession number MSV000094477. The RNA-seq data are available at the Sequence Read Archive (SRA) database (BioProject: PRJNA1028550).

## SUPPLEMENTAL MATERIAL

Figure S1 msystems.01226-23-s0001.tif Growth curves up to 72 h of the environmental ATCC30010 (Neff) and clinical ATCC50370 (T4) strains of *A. castellanii*.

Figure S2 msystems.01226-23-s0002.tif Percentage of enriched proteins based on GO functions found in EVs of environmental Neff and clinical T4 strains of *A. castellanii*.

Figure S3 msystems.01226-23-s0003.tif Lipid enrichment analysis of the differentially regulated species found in EVs of environmental Neff and clinical T4 strains of *A. castellanii*.

Figure S4 msystems.01226-23-s0004.tif Differentially regulated proteins more abundant in Neff EVs and T4 EVs that participate in the aminoacyl-tRNA biosynthesis and regulation of protein expression/translation.

Figure S5 msystems.01226-23-s0005.tif Differentially abundant proteins involved in transport and catabolism found in Neff EVs and T4 EVs.

Table S1 msystems.01226-23-s0006.xlsx Proteins detected by proteomics analysis in the EVs of Neff and T4 strains of *A. castellanii*.

Table S2 msystems.01226-23-s0007.xlsx Lipid species found in the lipidomic positive mode analysis of EV from Neff and T4 strains of *A. castellanii*.

Table S3 msystems.01226-23-s0008.xlsx Lipid species found in the lipidomic negative mode analysis of EV from Neff and T4 strains of *A. castellanii*.

Table S4 msystems.01226-23-s0009.xlsx Metabolites found in the metabolomics analysis of EV from Neff and T4 strains of *A. castellanii*.

ASM does not own the copyrights to Supplemental Material that may be linked to, or accessed through, an article. The authors have granted ASM a non-exclusive, world-wide license to publish the Supplemental Material files. Please contact the corresponding author directly for reuse.

## References

[1] Fu MS, Liporagi-Lopes LC, Dos Santos SR, Tenor JL, Perfect JR, Cuomo CA, Casadevall A. 2021. Amoeba predation of Cryptococcus neoformans results in pleiotropic changes to traits associated with virulence. mBio 12:e00567-21. doi:10.1128/mBio.00567-21 33906924 PMC8092252

[2] Steenbergen JN, Shuman HA, Casadevall A. 2001. Cryptococcus neoformans interactions with amoebae suggest an explanation for its virulence and intracellular pathogenic strategy in macrophages. Proc Natl Acad Sci U S A 98:15245–15250. doi:10.1073/pnas.261418798 11742090 PMC65014

[3] Gonçalves D de S, Ferreira M da S, Gomes KX, Rodríguez-de La Noval C, Liedke SC, da Costa GCV, Albuquerque P, Cortines JR, Saramago Peralta RH, Peralta JM, Casadevall A, Guimarães AJ. 2019. Unravelling the interactions of the environmental host Acanthamoeba castellanii with fungi through the recognition by mannose-binding proteins. Cell Microbiol 21:e13066. doi:10.1111/cmi.13066 31173452

[4] Guimaraes AJ, Gomes KX, Cortines JR, Peralta JM, Peralta RHS. 2016. Acanthamoeba spp. as a universal host for pathogenic microorganisms: one bridge from environment to host virulence. Microbiol Res 193:30–38. doi:10.1016/j.micres.2016.08.001 27825484

[5] Mungroo MR, Siddiqui R, Khan NA. 2021. War of the microbial world: Acanthamoeba spp. interactions with microorganisms. Folia Microbiol (Praha) 66:689–699. doi:10.1007/s12223-021-00889-7 34145552 PMC8212903

[6] Goy G, Thomas V, Rimann K, Jaton K, Prod’hom G, Greub G. 2007. The Neff strain of Acanthamoeba castellanii, a tool for testing the virulence of Mycobacterium kansasii. Res Microbiol 158:393–397. doi:10.1016/j.resmic.2007.01.003 17398074

[7] da Silva Ferreira M, de Souza Gonçalves D, Medeiros EG, Peralta JM, Guimarães AJ. 2021. "Feast-fit-fist-feat": overview of free-living amoeba interactions with fungi and virulence as a foundation for success in battle. Curr Trop Med Rep 8:18–31. doi:10.1007/s40475-020-00220-3

[8] Casadevall A, Fu M, Guimaraes A, Albuquerque P. 2019. The ‘amoeboid predator-fungal animal virulence’ hypothesis. J Fungi 5:10. doi:10.3390/jof5010010 PMC6463022 30669554

[9] Ferreira M da S, Mendoza SR, Gonçalves D de S, Rodríguez-de la Noval C, Honorato L, Nimrichter L, Ramos LFC, Nogueira FCS, Domont GB, Peralta JM, Guimarães AJ. 2022. Recognition of cell wall mannosylated components as a conserved feature for fungal entrance, adaptation and survival within trophozoites of Acanthamoeba castellanii and murine macrophages. Front Cell Infect Microbiol 12:858979. doi:10.3389/fcimb.2022.858979 35711659 PMC9194641

[10] Siddiqui R, Khan NA. 2012. Acanthamoeba is an evolutionary ancestor of macrophages: a myth or reality? Exp Parasitol 130:95–97. doi:10.1016/j.exppara.2011.11.005 22143089

[11] Jeong HJ, Jang ES, Han BI, Lee KH, Ock MS, Kong HH, Chung DI, Seol SY, Cho DT, Yu HS. 2007. Acanthamoeba: could it be an environmental host of Shigella?. Exp Parasitol 115:181–186. doi:10.1016/j.exppara.2006.08.002 16978610

[12] Walochnik J, Scheikl U, Haller-Schober E-M. 2015. Twenty years of Acanthamoeba diagnostics in Austria. J Eukaryot Microbiol 62:3–11. doi:10.1111/jeu.12149 25047131 PMC4342769

[13] Diehl MLN, Paes J, Rott MB. 2021. Genotype distribution of Acanthamoeba in keratitis: a systematic review. Parasitol Res 120:3051–3063. doi:10.1007/s00436-021-07261-1 34351492 PMC8339388

[14] Siddiqui R, Khan NA. 2012. Biology and pathogenesis of Acanthamoeba. Parasit Vectors 5:6. doi:10.1186/1756-3305-5-6 22229971 PMC3284432

[15] Connelly L, Anijeet D, Alexander CL. 2020. A descriptive case of persistent Acanthamoeba keratitis: raising awareness of this complex ocular disease. Access Microbiol 2:acmi000084. doi:10.1099/acmi.0.000084 32974565 PMC7470307

[16] Visvesvara GS. 2013. Infections with free-living amebae, p. 153–168. Handbook of clinical neurology. Elsevier. 10.1016/B978-0-444-53490-3.00010-8 23829906

[17] Zamora A, Henderson H, Swiatlo E. 2014. Acanthamoeba encephalitis: a case report and review of therapy. Surg Neurol Int 5:68. doi:10.4103/2152-7806.132239 24991471 PMC4078452

[18] Corsaro D, Köhsler M, Venditti D, Rott MB, Walochnik J. 2019. Recovery of an Acanthamoeba strain with two group I Introns in the nuclear 18S rRNA gene. Eur J Protistol 68:88–98. doi:10.1016/j.ejop.2019.01.007 30743186

[19] Alves D de SMM, Moraes AS, Alves LM, Gurgel-Gonçalves R, Lino Junior R de S, Cuba-Cuba CA, Vinaud MC. 2016. Experimental infection of t4 Acanthamoeba genotype determines the pathogenic potential. Parasitol Res 115:3435–3440. doi:10.1007/s00436-016-5105-3 27164833

[20] Kot K, Łanocha-Arendarczyk NA, Kosik-Bogacka DI. 2018. Amoebas from the genus Acanthamoeba and their pathogenic properties. Ann Parasitol 64:299–308. doi:10.17420/ap6404.164 30720249

[21] Mirjalali H, Niyyati M, Abedkhojasteh H, Babaei Z, Sharifdini M, Rezaeian M. 2013. Pathogenic assays of Acanthamoeba belonging to the t4 genotype. Iran J Parasitol 8:530–535. 25516733 PMC4266116

[22] Gu X, Lu X, Lin S, Shi X, Shen Y, Lu Q, Yang Y, Yang J, Cai J, Fu C, Lou Y, Zheng M. 2022. A comparative genomic approach to determine the virulence factors and horizontal gene transfer events of clinical Acanthamoeba isolates. Microbiol Spectr 10:e0002522. doi:10.1128/spectrum.00025-22 35416714 PMC9045148

[23] Wang Y, Jiang L, Zhao Y, Ju X, Wang L, Jin L, Fine RD, Li M. 2023. Biological characteristics and pathogenicity of Acanthamoeba. Front Microbiol 14:1147077. doi:10.3389/fmicb.2023.1147077 37089530 PMC10113681

[24] Schuster FL, Visvesvara GS. 2004. Free-living amoebae as opportunistic and non-opportunistic pathogens of humans and animals. Int J Parasitol 34:1001–1027. doi:10.1016/j.ijpara.2004.06.004 15313128

[25] Reyes-Batlle M, Mura-Escorche G, Sifaoui I, Otero-Ruiz A, Alfaro-Sifuentes R, López-Arencibia A, Rocha-Cabrera P, Chiboub O, Rizo-Liendo A, Zamora-Herrera J, Bethencourt-Estrella CJ, Rodríguez-Expósito RL, Nicolás-Hernández DS, Piñero JE, Lorenzo-Morales J. 2019. In vitro evaluation of combined commercialized ophthalmic solutions against Acanthamoeba strains. Pathogens 8:109. doi:10.3390/pathogens8030109 31349717 PMC6789763

[26] Lahiri R, Krahenbuhl JL. 2008. The role of free-living pathogenic amoeba in the transmission of leprosy: a proof of principle. Lepr Rev 79:401–409. 19274986

[27] Garate M, Cao Z, Bateman E, Panjwani N. 2004. Cloning and characterization of a novel mannose-binding protein of Acanthamoeba. J Biol Chem 279:29849–29856. doi:10.1074/jbc.M402334200 15117936

[28] Rocha-Azevedo B da, Jamerson M, Cabral GA, Marciano-Cabral F. 2010. Acanthamoeba culbertsoni: analysis of amoebic adhesion and invasion on extracellular matrix components collagen I and laminin-1. Exp Parasitol 126:79–84. doi:10.1016/j.exppara.2009.08.004 19698710

[29] Chusattayanond AD, Boonsilp S, Kasisit J, Boonmee A, Warit S. 2010. Thai Acanthamoeba isolate (T4) induced apoptotic death in neuroblastoma cells via the bax-mediated pathway. Parasitol Int 59:512–516. doi:10.1016/j.parint.2010.06.007 20601106

[30] Pellegrin JL, Ortega-Barria E, Prioli RP, Meijia JS, Pereira ME. 1992. The neuraminidases of Trypanosoma cruzi and Acanthamoeba castellanii are immunologically related. Trop Med Parasitol Off Organ Dtsch Tropenmedizinische Ges Dtsch Ges Tech Zusammenarbeit GTZ 43:33–37. 1376002

[31] Choi DH, Na BK, Seo MS, Song HR, Song CY. 2000. Purification and characterization of iron superoxide dismutase and copper-zinc superoxide dismutase from Acanthamoeba castellanii. J Parasitol 86:899–907. doi:10.1645/0022-3395(2000)086[0899:PACOIS]2.0.CO;2 11128508

[32] Khan NA. 2015. Acanthamoeba: biology and pathogenesis. Caister Academic Press, Karachi, Pakistan. Available from: http://www.horizonpress.com/acanthamoeba2. Retrieved 24 Jul 2023.

[33] Matin A, Jung S-Y. 2011. Phospholipase activities in clinical and environmental isolates of Acanthamoeba. Korean J Parasitol 49:1–8. doi:10.3347/kjp.2011.49.1.1 21461262 PMC3063920

[34] Mortazavi PN, Keisary E, Loh LN, Jung S-Y, Khan NA. 2011. Possible roles of phospholipase A(2) in the biological activities of Acanthamoeba castellanii (T4 genotype). Protist 162:168–176. doi:10.1016/j.protis.2010.03.005 20650684

[35] Khan NA, Jarroll EL, Panjwani N, Cao Z, Paget TA. 2000. Proteases as markers for differentiation of pathogenic and nonpathogenic species of Acanthamoeba. J Clin Microbiol 38:2858–2861. doi:10.1128/JCM.38.8.2858-2861.2000 10921939 PMC87129

[36] Gonçalves D de S, Ferreira M da S, Guimarães AJ. 2019. Extracellular vesicles from the protozoa Acanthamoeba castellanii: their role in pathogenesis, environmental adaptation and potential applications. Bioengineering (Basel) 6:13. doi:10.3390/bioengineering6010013 30717103 PMC6466093

[37] Gonçalves D de S, Ferreira M da S, Liedke SC, Gomes KX, de Oliveira GA, Leão PEL, Cesar GV, Seabra SH, Cortines JR, Casadevall A, Nimrichter L, Domont GB, Junqueira MR, Peralta JM, Guimaraes AJ. 2018. Extracellular vesicles and vesicle-free secretome of the protozoa Acanthamoeba castellanii under homeostasis and nutritional stress and their damaging potential to host cells. Virulence 9:818–836. doi:10.1080/21505594.2018.1451184 29560793 PMC5955443

[38] Omaña-Molina M, González-Robles A, Iliana Salazar-Villatoro L, Lorenzo-Morales J, Cristóbal-Ramos AR, Hernández-Ramírez VI, Talamás-Rohana P, Méndez Cruz AR, Martínez-Palomo A. 2013. Reevaluating the role of Acanthamoeba proteases in tissue invasion: observation of cytopathogenic mechanisms on MDCK cell monolayers and hamster corneal cells. Biomed Res Int 2013:461329. doi:10.1155/2013/461329 23484119 PMC3581277

[39] Lin W-C, Tsai C-Y, Huang J-M, Wu S-R, Chu LJ, Huang K-Y. 2019. Quantitative proteomic analysis and functional characterization of Acanthamoeba castellanii exosome-like vesicles. Parasit Vectors 12:467. doi:10.1186/s13071-019-3725-z 31597577 PMC6784334

[40] Retana Moreira L, Vargas Ramírez D, Linares F, Prescilla Ledezma A, Vaglio Garro A, Osuna A, Lorenzo Morales J, Abrahams Sandí E. 2020. Isolation of Acanthamoeba T5 from water: characterization of its pathogenic potential, including the production of extracellular vesicles. Pathogens 9:144. doi:10.3390/pathogens9020144 32098034 PMC7168589

[41] Costa AO, Chagas IAR, de Menezes-Neto A, Rêgo FD, Nogueira PM, Torrecilhas AC, Furst C, Fux B, Soares RP. 2021. Distinct immunomodulatory properties of extracellular vesicles released by different strains of Acanthamoeba. Cell Biol Int 45:1060–1071. doi:10.1002/cbin.11551 33448518

[42] Nakayasu ES, Nicora CD, Sims AC, Burnum-Johnson KE, Kim Y-M, Kyle JE, Matzke MM, Shukla AK, Chu RK, Schepmoes AA, Jacobs JM, Baric RS, Webb-Robertson B-J, Smith RD, Metz TO. 2016. MPLEx: a robust and universal protocol for single-sample integrative proteomic, metabolomic, and lipidomic analyses. mSystems 1:e00043-16. doi:10.1128/mSystems.00043-16 27822525 PMC5069757

[43] Liu J. 2008. Control of protein synthesis and mRNA degradation by microRNAs. Curr Opin Cell Biol 20:214–221. doi:10.1016/j.ceb.2008.01.006 18329869

[44] Zamith-Miranda D, Heyman HM, Couvillion SP, Cordero RJB, Rodrigues ML, Nimrichter L, Casadevall A, Amatuzzi RF, Alves LR, Nakayasu ES, Nosanchuk JD. 2021. Comparative molecular and immunoregulatory analysis of extracellular vesicles from Candida albicans and Candida auris. mSystems 6:e00822–21. doi:10.1128/mSystems.00822-21 34427507 PMC8407381

[45] Coelho C, Brown L, Maryam M, Vij R, Smith DFQ, Burnet MC, Kyle JE, Heyman HM, Ramirez J, Prados-Rosales R, Lauvau G, Nakayasu ES, Brady NR, Hamacher-Brady A, Coppens I, Casadevall A. 2019. Listeria monocytogenes virulence factors, including listeriolysin O, are secreted in biologically active extracellular vesicles. J Biol Chem 294:1202–1217. doi:10.1074/jbc.RA118.006472 30504226 PMC6349127

[46] Zhang L, Zhang K, Li H, Coelho C, de Souza Gonçalves D, Fu MS, Li X, Nakayasu ES, Kim Y-M, Liao W, Pan W, Casadevall A. 2021. Cryptococcus neoformans - infected macrophages release proinflammatory extracellular vesicles: insight into their components by multi-omics. mBio 12:e00279-21. doi:10.1128/mBio.00279-21 33785616 PMC8092229

[47] Witwer KW, Goberdhan DC, O’Driscoll L, Théry C, Welsh JA, Blenkiron C, Buzás EI, Di Vizio D, Erdbrügger U, Falcón-Pérez JM, Fu Q-L, Hill AF, Lenassi M, Lötvall J, Nieuwland R, Ochiya T, Rome S, Sahoo S, Zheng L. 2021. Updating MISEV: evolving the minimal requirements for studies of extracellular vesicles. J Extracell Vesicles 10:e12182. doi:10.1002/jev2.12182 34953156 PMC8710080

[48] Théry C, Witwer KW, Aikawa E, Alcaraz MJ, Anderson JD, Andriantsitohaina R, Antoniou A, Arab T, Archer F, Atkin‐Smith GK, et al.. 2018. Minimal information for studies of extracellular vesicles 2018 (MISEV2018): a position statement of the international society for extracellular vesicles and update of the MISEV2014 guidelines. J of Extracellular Vesicle 7:1535750. doi:10.1080/20013078.2018.1535750 PMC6322352 30637094

[49] Frases S, Pontes B, Nimrichter L, Viana NB, Rodrigues ML, Casadevall A. 2009. Capsule of Cryptococcus neoformans grows by enlargement of polysaccharide molecules. Proc Natl Acad Sci U S A 106:1228–1233. doi:10.1073/pnas.0808995106 19164571 PMC2633523

[50] Matos Baltazar L, Nakayasu ES, Sobreira TJP, Choi H, Casadevall A, Nimrichter L, Nosanchuk JD. 2016. Antibody binding alters the characteristics and contents of extracellular vesicles released by Histoplasma capsulatum. mSphere 1:e00085-15. doi:10.1128/mSphere.00085-15 PMC4894687 27303729

[51] Zamith-Miranda D, Heyman HM, Cleare LG, Couvillion SP, Clair GC, Bredeweg EL, Gacser A, Nimrichter L, Nakayasu ES, Nosanchuk JD. 2019. Multi-omics signature of Candida auris, an emerging and multidrug-resistant pathogen. mSystems 4:e00257-19. doi:10.1128/mSystems.00257-19 31186339 PMC6561322

[52] Cox J, Mann M. 2008. Maxquant enables high peptide identification rates, individualized p. p.b.-range mass accuracies and proteome-wide protein quantification. Nat Biotechnol 26:1367–1372. doi:10.1038/nbt.1511 19029910

[53] Kyle JE, Crowell KL, Casey CP, Fujimoto GM, Kim S, Dautel SE, Smith RD, Payne SH, Metz TO. 2017. LIQUID: an-open source software for identifying lipids in LC-MS/MS-based lipidomics data. Bioinformatics 33:1744–1746. doi:10.1093/bioinformatics/btx046 28158427 PMC5860051

[54] Pluskal T, Castillo S, Villar-Briones A, Oresic M. 2010. MZmine 2: modular framework for processing, visualizing, and analyzing mass spectrometry-based molecular profile data. BMC Bioinformatics 11:395. doi:10.1186/1471-2105-11-395 20650010 PMC2918584

[55] Hiller K, Hangebrauk J, Jäger C, Spura J, Schreiber K, Schomburg D. 2009. MetaboliteDetector: comprehensive analysis tool for targeted and nontargeted GC/MS based metabolome analysis. Anal Chem 81:3429–3439. doi:10.1021/ac802689c 19358599

[56] Kind T, Wohlgemuth G, Lee DY, Lu Y, Palazoglu M, Shahbaz S, Fiehn O. 2009. FiehnLib: mass spectral and retention index libraries for metabolomics based on quadrupole and time-of-flight gas chromatography/mass spectrometry. Anal Chem 81:10038–10048. doi:10.1021/ac9019522 19928838 PMC2805091

[57] Sherman BT, Hao M, Qiu J, Jiao X, Baseler MW, Lane HC, Imamichi T, Chang W. 2022. DAVID: a web server for functional enrichment analysis and functional annotation of gene lists (2021 update). Nucleic Acids Res 50:W216–W221. doi:10.1093/nar/gkac194 35325185 PMC9252805

[58] Hosack DA, Dennis G, Sherman BT, Lane HC, Lempicki RA. 2003. Identifying biological themes within lists of genes with EASE. Genome Biol 4:R70. doi:10.1186/gb-2003-4-10-r70 14519205 PMC328459

[59] Clair G, Bramer LM, Misra R, McGraw MD, Bhattacharya S, Kitzmiller JA, Feng S, Danna VG, Bandyopadhyay G, Bhotika H, Huyck HL, Deutsch GH, Mariani TJ, Carson JP, Whitsett JA, Pryhuber GS, Adkins JN, Ansong C. 2022. Proteomic analysis of human lung development. Am J Respir Crit Care Med 205:208–218. doi:10.1164/rccm.202008-3303OC 34752721 PMC8787240

[60] Huang DW, Sherman BT, Lempicki RA. 2009. Systematic and integrative analysis of large gene lists using DAVID bioinformatics resources. Nat Protoc 4:44–57. doi:10.1038/nprot.2008.211 19131956

[61] Rohn H, Junker A, Hartmann A, Grafahrend-Belau E, Treutler H, Klapperstück M, Czauderna T, Klukas C, Schreiber F. 2012. VANTED v2: a framework for systems biology applications. BMC Syst Biol 6:139. doi:10.1186/1752-0509-6-139 23140568 PMC3610154

[62] Clair G, Reehl S, Stratton KG, Monroe ME, Tfaily MM, Ansong C, Kyle JE. 2019. Lipid mini-on: mining and ontology tool for enrichment analysis of lipidomic data. Bioinformatics 35:4507–4508. doi:10.1093/bioinformatics/btz250 30977807 PMC7963073

[63] Chong J, Soufan O, Li C, Caraus I, Li S, Bourque G, Wishart DS, Xia J. 2018. MetaboAnalyst 4.0: towards more transparent and integrative metabolomics analysis. Nucleic Acids Res 46:W486–W494. doi:10.1093/nar/gky310 29762782 PMC6030889

[64] Willms E, Cabañas C, Mäger I, Wood MJA, Vader P. 2018. Extracellular vesicle heterogeneity: subpopulations, isolation techniques, and diverse functions in cancer progression. Front Immunol 9:738. doi:10.3389/fimmu.2018.00738 29760691 PMC5936763

[65] Welsh JA, Goberdhan DCI, O’Driscoll L, Buzas EI, Blenkiron C, Bussolati B, Cai H, Di Vizio D, Driedonks TAP, Erdbrügger U, et al.. 2024. Minimal information for studies of extracellular vesicles (MISEV2023): from basic to advanced approaches. J of Extracellular Vesicle 13. doi:10.1002/jev2.12404 PMC10850029 38326288

[66] Rodrigues ML, Casadevall A. 2018. A two-way road: novel roles for fungal extracellular vesicles. Mol Microbiol 110:11–15. doi:10.1111/mmi.14095 30079549

[67] Raposo G, Stoorvogel W. 2013. Extracellular vesicles: exosomes, microvesicles, and friends. J Cell Biol 200:373–383. doi:10.1083/jcb.201211138 23420871 PMC3575529

[68] Toyofuku M, Morinaga K, Hashimoto Y, Uhl J, Shimamura H, Inaba H, Schmitt-Kopplin P, Eberl L, Nomura N. 2017. Membrane vesicle-mediated bacterial communication. ISME J 11:1504–1509. doi:10.1038/ismej.2017.13 28282039 PMC5437348

[69] Fu H, Elena RC, Marquez PH. 2019. The roles of small RNAs: insights from bacterial quorum sensing. ExRNA 1:32. doi:10.1186/s41544-019-0027-8

[70] Geiger A, Hirtz C, Bécue T, Bellard E, Centeno D, Gargani D, Rossignol M, Cuny G, Peltier J-B. 2010. Exocytosis and protein secretion in Trypanosoma. BMC Microbiol 10:20. doi:10.1186/1471-2180-10-20 20102621 PMC3224696

[71] Marti M, Johnson PJ. 2016. Emerging roles for extracellular vesicles in parasitic infections. Curr Opin Microbiol 32:66–70. doi:10.1016/j.mib.2016.04.008 27208506 PMC6445373

[72] Buck AH, Coakley G, Simbari F, McSorley HJ, Quintana JF, Le Bihan T, Kumar S, Abreu-Goodger C, Lear M, Harcus Y, Ceroni A, Babayan SA, Blaxter M, Ivens A, Maizels RM. 2014. Exosomes secreted by nematode parasites transfer small RNAs to mammalian cells and modulate innate immunity. Nat Commun 5:5488. doi:10.1038/ncomms6488 25421927 PMC4263141

[73] Marcilla A, Trelis M, Cortés A, Sotillo J, Cantalapiedra F, Minguez MT, Valero ML, Sánchez del Pino MM, Muñoz-Antoli C, Toledo R, Bernal D. 2012. Extracellular vesicles from parasitic helminths contain specific excretory/secretory proteins and are internalized in intestinal host cells. PLoS One 7:e45974. doi:10.1371/journal.pone.0045974 23029346 PMC3454434

[74] Tatischeff I. 2019. Dictyostelium: a model for studying the extracellular vesicle messengers involved in human health and disease. Cells 8:225. doi:10.3390/cells8030225 30857191 PMC6468606

[75] Tatischeff I. 2013. Assets of the non-pathogenic microorganism Dictyostelium discoideum as a model for the study of eukaryotic extracellular vesicles. F1000Res 2:73. doi:10.12688/f1000research.2-73.v1 PMC3782363 24327885

[76] Sierra-López F, Castelan-Ramírez I, Hernández-Martínez D, Salazar-Villatoro L, Segura-Cobos D, Flores-Maldonado C, Hernández-Ramírez VI, Villamar-Duque TE, Méndez-Cruz AR, Talamás-Rohana P, Omaña-Molina M. 2023. Extracellular vesicles secreted by Acanthamoeba culbertsoni have COX and proteolytic activity and induce hemolysis. Microorganisms 11:2762. doi:10.3390/microorganisms11112762 38004773 PMC10673465

[77] Lertjuthaporn S, Somkird J, Lekmanee K, Atipimonpat A, Sukapirom K, Sawasdipokin H, Tiewcharoen S, Pattanapanyasat K, Khowawisetsut L. 2022. Extracellular vesicles from Naegleria fowleri induce IL-8 response in THP-1 macrophage. Pathogens 11:632. doi:10.3390/pathogens11060632 35745486 PMC9231210

[78] Retana Moreira L, Steller Espinoza MF, Chacón Camacho N, Cornet-Gomez A, Sáenz-Arce G, Osuna A, Lomonte B, Abrahams Sandí E. 2022. Characterization of extracellular vesicles secreted by a clinical isolate of Naegleria fowleri and identification of immunogenic components within their protein cargo. Biology (Basel) 11:983. doi:10.3390/biology11070983 36101365 PMC9312180

[79] Russell AC, Bush P, Grigorean G, Kyle DE. 2023. Characterization of the extracellular vesicles, ultrastructural morphology, and intercellular interactions of multiple clinical isolates of the brain-eating amoeba, Naegleria fowleri. Front Microbiol 14:1264348. doi:10.3389/fmicb.2023.1264348 37808283 PMC10558758

[80] Sharma M, Morgado P, Zhang H, Ehrenkaufer G, Manna D, Singh U. 2020. Characterization of extracellular vesicles from entamoeba histolytica identifies roles in intercellular communication that regulates parasite growth and development. Infect Immun 88:e00349–20. doi:10.1128/IAI.00349-20 32719158 PMC7504958

[81] Szempruch AJ, Dennison L, Kieft R, Harrington JM, Hajduk SL. 2016. Sending a message: extracellular vesicles of pathogenic protozoan parasites. Nat Rev Microbiol 14:669–675. doi:10.1038/nrmicro.2016.110 27615028

[82] Tzelos T, Matthews JB, Buck AH, Simbari F, Frew D, Inglis NF, McLean K, Nisbet AJ, Whitelaw CBA, Knox DP, McNeilly TN. 2016. A preliminary proteomic characterisation of extracellular vesicles released by the ovine parasitic nematode, teladorsagia circumcincta. Vet Parasitol 221:84–92. doi:10.1016/j.vetpar.2016.03.008 27084478 PMC4867787

[83] Chaiyadet S, Sotillo J, Smout M, Cantacessi C, Jones MK, Johnson MS, Turnbull L, Whitchurch CB, Potriquet J, Laohaviroj M, Mulvenna J, Brindley PJ, Bethony JM, Laha T, Sripa B, Loukas A. 2015. Carcinogenic liver fluke secretes extracellular vesicles that promote cholangiocytes to adopt a tumorigenic phenotype. J Infect Dis 212:1636–1645. doi:10.1093/infdis/jiv291 25985904 PMC4621255

[84] Tatischeff I, Lavialle F, Pigaglio-Deshayes S, Péchoux-Longin C, Chinsky L, Alfsen A. 2008. Dictyostelium extracellular vesicles containing hoechst 33342 transfer the dye into the nuclei of living cells: a fluorescence study. J Fluoresc 18:319–328. doi:10.1007/s10895-007-0271-4 18074206

[85] Bachurski D, Schuldner M, Nguyen P-H, Malz A, Reiners KS, Grenzi PC, Babatz F, Schauss AC, Hansen HP, Hallek M, Pogge von Strandmann E. 2019. Extracellular vesicle measurements with nanoparticle tracking analysis - an accuracy and repeatability comparison between NanoSight NS300 and ZetaView. J Extracell Vesicles 8:1596016. doi:10.1080/20013078.2019.1596016 30988894 PMC6450530

[86] Bryant G, Abeynayake C, Thomas JC. 1996. Improved particle size distribution measurements using multiangle dynamic light scattering. 2. refinements and applications. Langmuir 12:6224–6228. doi:10.1021/la960224o

[87] Skliar M, Chernyshev VS, Belnap DM, Sergey GV, Al-Hakami SM, Bernard PS, Stijleman IJ, Rachamadugu R. 2018. Membrane proteins significantly restrict exosome mobility. Biochem Biophys Res Commun 501:1055–1059. doi:10.1016/j.bbrc.2018.05.107 29777705

[88] Loomis WF. 2014. Cell signaling during development of Dictyostelium. Dev Biol 391:1–16. doi:10.1016/j.ydbio.2014.04.001 24726820 PMC4075484

[89] Du Q, Kawabe Y, Schilde C, Chen Z-H, Schaap P. 2015. The evolution of aggregative multicellularity and cell–cell communication in the Dictyostelia. J Mol Biol 427:3722–3733. doi:10.1016/j.jmb.2015.08.008 26284972 PMC5055082

[90] Dudley R, Alsam S, Khan NA. 2008. The role of proteases in the differentiation of Acanthamoeba castellanii. FEMS Microbiol Lett 286:9–15. doi:10.1111/j.1574-6968.2008.01249.x 18616591

[91] Mahdavi Poor B, Dalimi A, Ghafarifar F, Khoshzaban F, Abdolalizadeh J. 2017. Characterization of extracellular proteases of Acanthamoeba genotype T4 isolated from different sources in Iran. Parasitol Res 116:3373–3380. doi:10.1007/s00436-017-5656-y 29075925

[92] Rocha-Azevedo BD, Jamerson M, Cabral GA, Silva-Filho FC, Marciano-Cabral F. 2009. Acanthamoeba interaction with extracellular matrix glycoproteins: biological and biochemical characterization and role in cytotoxicity and invasiveness. J Eukaryot Microbiol 56:270–278. doi:10.1111/j.1550-7408.2009.00399.x 19527355

[93] Nawaz M, Shah N, Zanetti BR, Maugeri M, Silvestre RN, Fatima F, Neder L, Valadi H. 2018. Extracellular vesicles and matrix remodeling enzymes: the emerging roles in extracellular matrix remodeling, progression of diseases and tissue repair. Cells 7:167. doi:10.3390/cells7100167 30322133 PMC6210724

[94] Burnum-Johnson KE, Kyle JE, Eisfeld AJ, Casey CP, Stratton KG, Gonzalez JF, Habyarimana F, Negretti NM, Sims AC, Chauhan S, et al.. 2017. MPLEx: a method for simultaneous pathogen inactivation and extraction of samples for multi-omics profiling. Analyst 142:442–448. doi:10.1039/C6AN02486F 28091625 PMC5283721

[95] Heidel AJ, Lawal HM, Felder M, Schilde C, Helps NR, Tunggal B, Rivero F, John U, Schleicher M, Eichinger L, Platzer M, Noegel AA, Schaap P, Glöckner G. 2011. Phylogeny-wide analysis of social amoeba genomes highlights ancient origins for complex Intercellular communication. Genome Res 21:1882–1891. doi:10.1101/gr.121137.111 21757610 PMC3205573

[96] Donoso-Quezada J, Ayala-Mar S, González-Valdez J. 2021. The role of lipids in exosome biology and intercellular communication: function, analytics and applications. Traffic 22:204–220. doi:10.1111/tra.12803 34053166 PMC8361711

[97] Carayon K, Chaoui K, Ronzier E, Lazar I, Bertrand-Michel J, Roques V, Balor S, Terce F, Lopez A, Salomé L, Joly E. 2011. Proteolipidic composition of exosomes changes during reticulocyte maturation. J Biol Chem 286:34426–34439. doi:10.1074/jbc.M111.257444 21828046 PMC3190795

[98] Beloribi S, Ristorcelli E, Breuzard G, Silvy F, Bertrand-Michel J, Beraud E, Verine A, Lombardo D. 2012. Exosomal lipids impact notch signaling and induce death of human pancreatic tumoral SOJ-6 cells. PLoS One 7:e47480. doi:10.1371/journal.pone.0047480 23094054 PMC3477155

[99] Hessvik NP, Llorente A. 2018. Current knowledge on exosome biogenesis and release. Cell Mol Life Sci 75:193–208. doi:10.1007/s00018-017-2595-9 28733901 PMC5756260

[100] Horbay R, Hamraghani A, Ermini L, Holcik S, Beug ST, Yeganeh B. 2022. Role of ceramides and lysosomes in extracellular vesicle biogenesis, cargo sorting and release. Int J Mol Sci 23:15317. doi:10.3390/ijms232315317 36499644 PMC9735581

[101] Cheng Q, Li X, Wang Y, Dong M, Zhan F-H, Liu J. 2018. The ceramide pathway is involved in the survival, apoptosis and exosome functions of human multiple myeloma cells in vitro. Acta Pharmacol Sin 39:561–568. doi:10.1038/aps.2017.118 28858294 PMC5888679

[102] Fitzner D, Schnaars M, van Rossum D, Krishnamoorthy G, Dibaj P, Bakhti M, Regen T, Hanisch U-K, Simons M. 2011. Selective transfer of exosomes from oligodendrocytes to microglia by macropinocytosis. J Cell Sci 124:447–458. doi:10.1242/jcs.074088 21242314

[103] Subra C, Laulagnier K, Perret B, Record M. 2007. Exosome lipidomics unravels lipid sorting at the level of multivesicular bodies. Biochimie 89:205–212. doi:10.1016/j.biochi.2006.10.014 17157973

[104] Laulagnier K, Motta C, Hamdi S, Roy S, Fauvelle F, Pageaux J-F, Kobayashi T, Salles J-P, Perret B, Bonnerot C, Record M. 2004. Mast cell- and dendritic cell-derived exosomes display a specific lipid composition and an unusual membrane organization. Biochem J 380:161–171. doi:10.1042/BJ20031594 14965343 PMC1224152

[105] Jeelani G, Nozaki T. 2014. Metabolomic analysis of entamoeba: applications and implications. Curr Opin Microbiol 20:118–124. doi:10.1016/j.mib.2014.05.016 24950028

[106] Zhang J, Li S, Li L, Li M, Guo C, Yao J, Mi S. 2015. Exosome and exosomal MicroRNA: trafficking, sorting, and function. Gen Pro Bio 13:17–24. doi:10.1016/j.gpb.2015.02.001 PMC4411500 25724326

[107] Valadi H, Ekström K, Bossios A, Sjöstrand M, Lee JJ, Lötvall JO. 2007. Exosome-mediated transfer of mRNAs and microRNAs is a novel mechanism of genetic exchange between cells. Nat Cell Biol 9:654–659. doi:10.1038/ncb1596 17486113

