# Supplementary figures and images for: Comprehensive characterization of extracellular vesicles produced by environmental (Neff) and clinical (T4) strains of Acanthamoeba castellanii
Source: mSystems. 2024 May 8;9(6):e01226-23. doi: 10.1128/msystems.01226-23 (PMC11237502; doi:10.1128/msystems.01226-23)

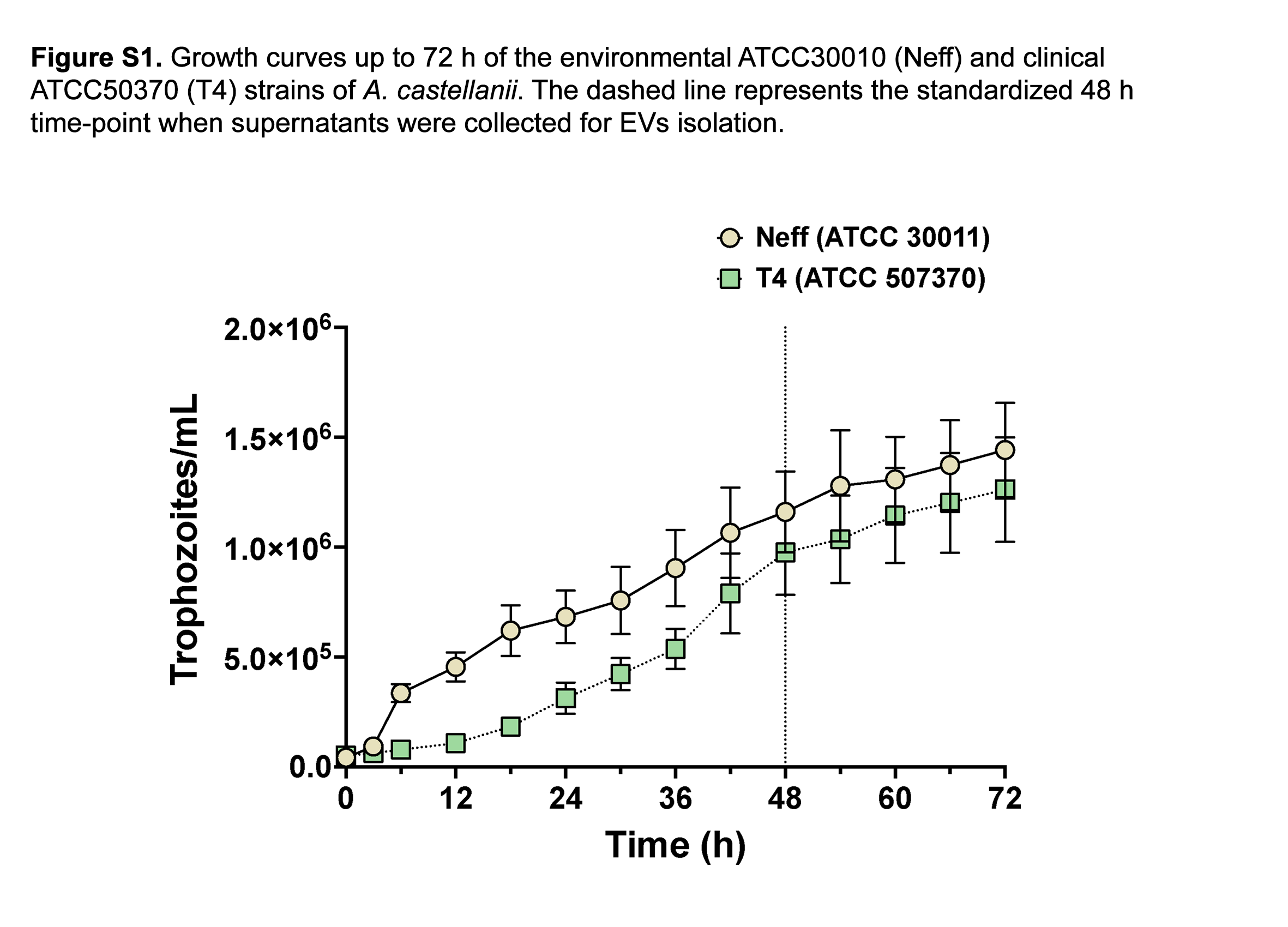

Supplement: Figure S1 — Growth curves up to 72 h of the environmental ATCC30010 (Neff) and clinical ATCC50370 (T4) strains of A. castellanii. [file msystems.01226-23-s0001.tif]

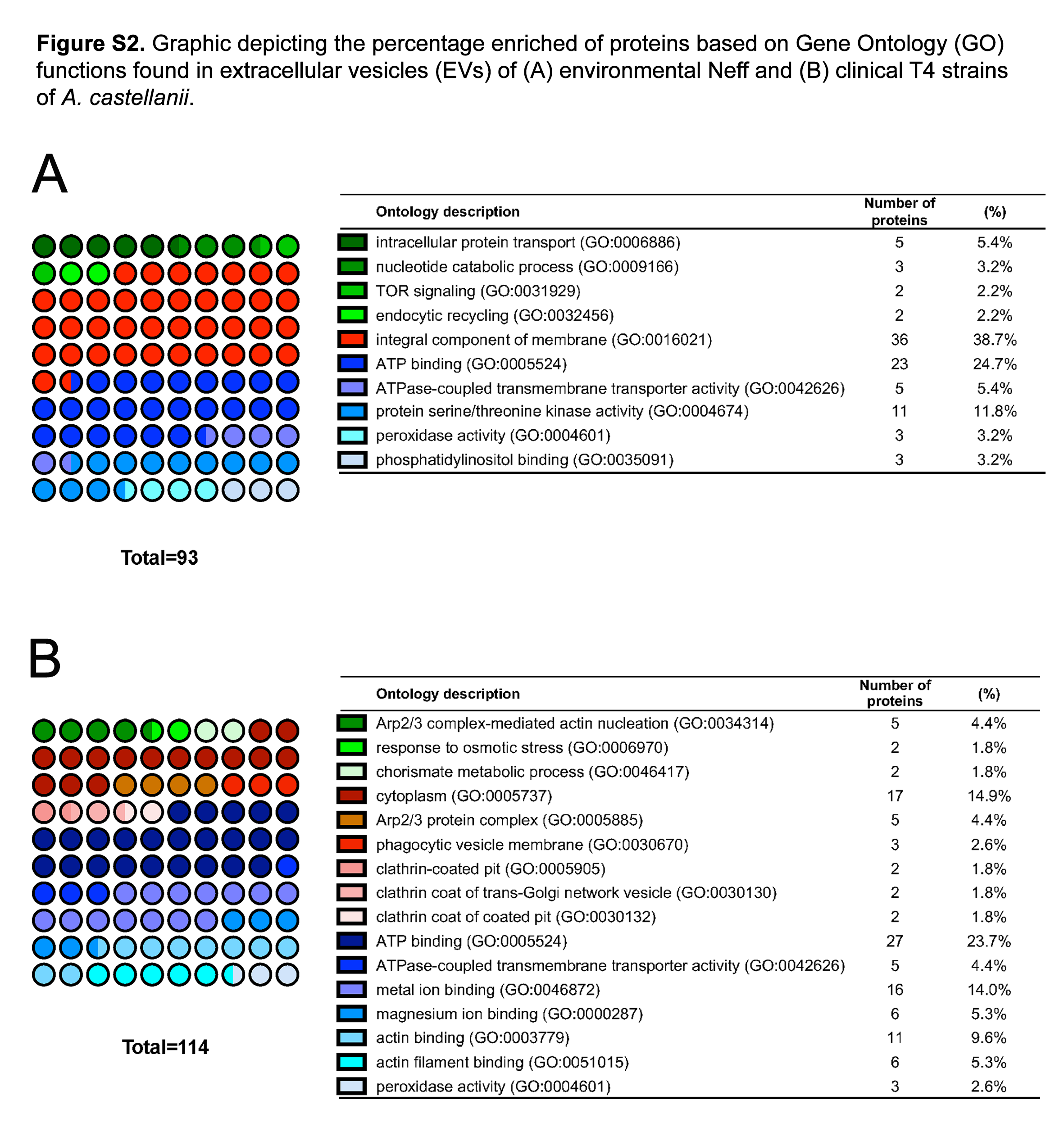

Supplement: Figure S2 — Percentage of enriched proteins based on GO functions found in EVs of environmental Neff and clinical T4 strains of A. castellanii. [file msystems.01226-23-s0002.tif]

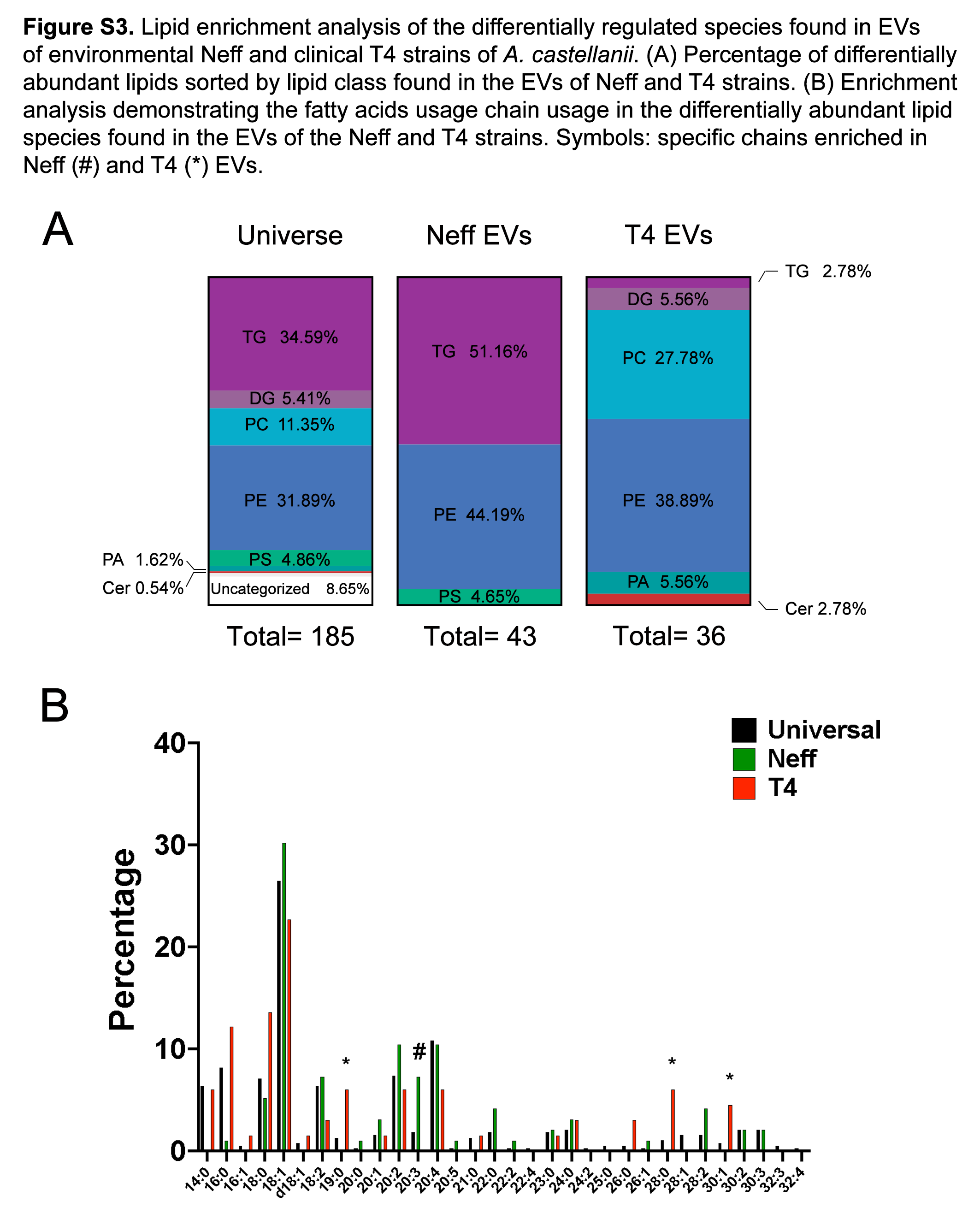

Supplement: Figure S3 — Lipid enrichment analysis of the differentially regulated species found in EVs of environmental Neff and clinical T4 strains of A. castellanii. [file msystems.01226-23-s0003.tif]

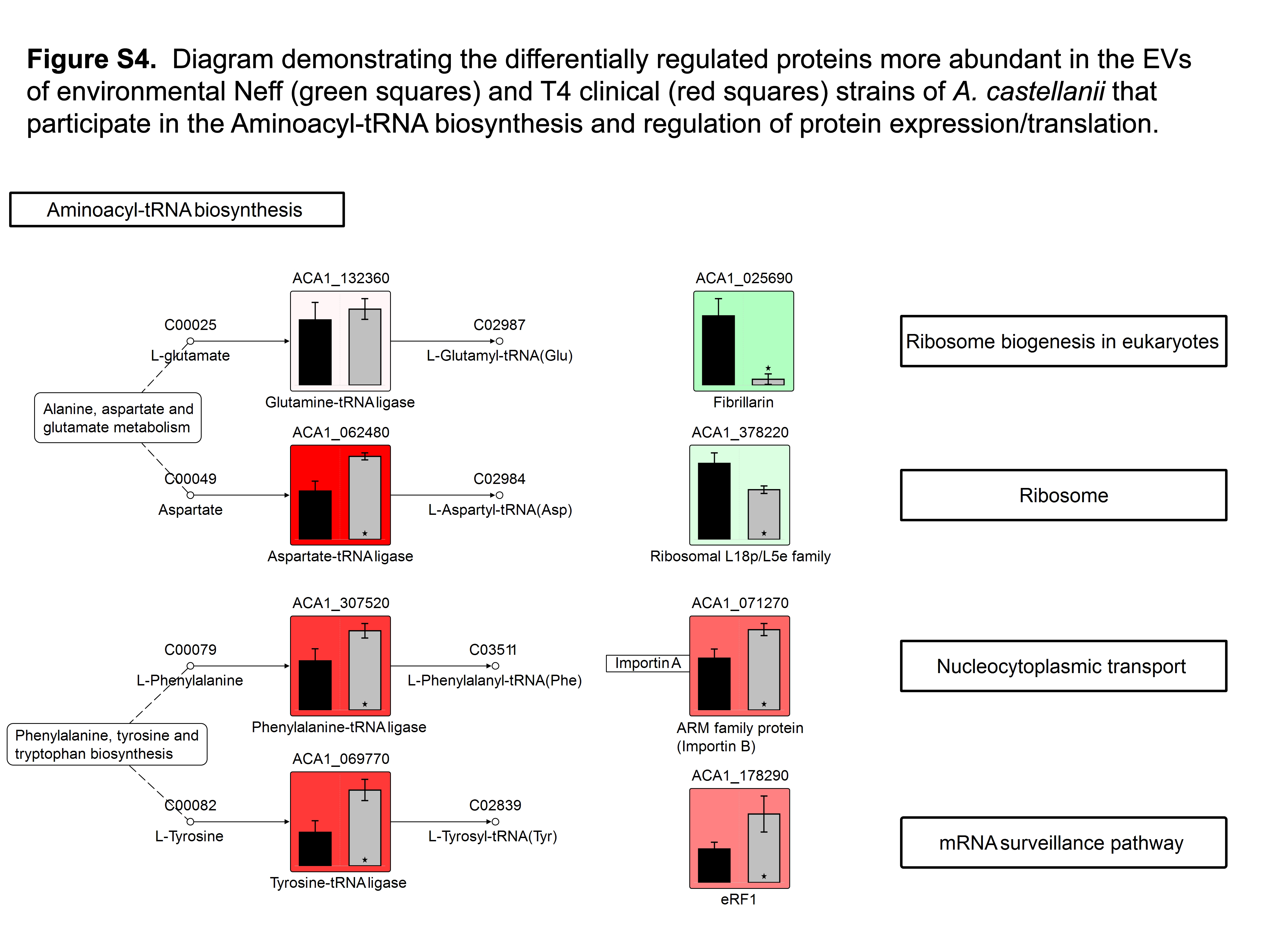

Supplement: Figure S4 — Differentially regulated proteins more abundant in Neff EVs and T4 EVs that participate in the aminoacyl-tRNA biosynthesis and regulation of protein expression/translation. [file msystems.01226-23-s0004.tif]

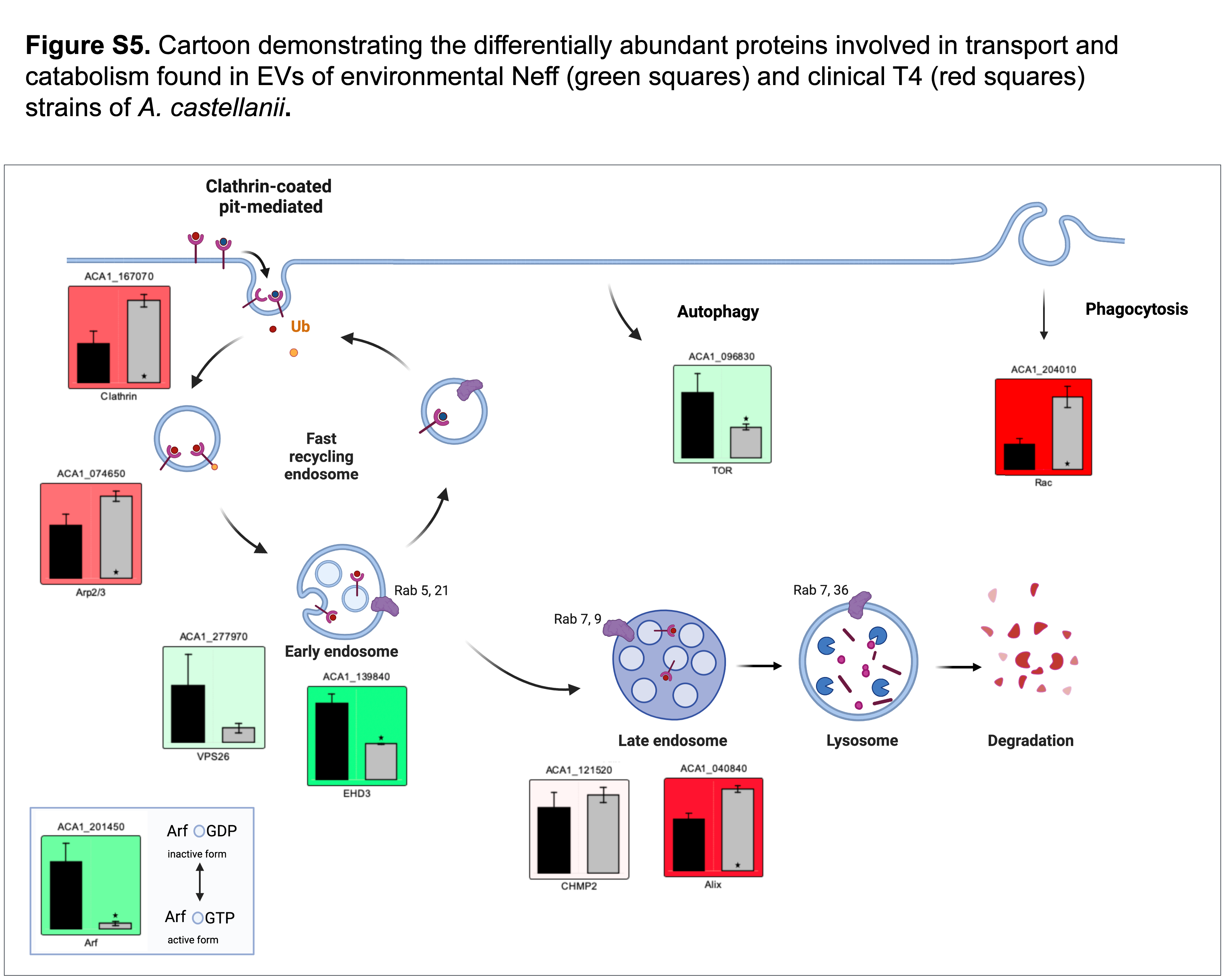

Supplement: Figure S5 — Differentially abundant proteins involved in transport and catabolism found in Neff EVs and T4 EVs. [file msystems.01226-23-s0005.tif]
